# The Ortholog Conjecture Is Untestable by the Current Gene Ontology but Is Supported by RNA Sequencing Data

**DOI:** 10.1371/journal.pcbi.1002784

**Published:** 2012-11-29

**Authors:** Xiaoshu Chen, Jianzhi Zhang

**Affiliations:** Department of Ecology and Evolutionary Biology, University of Michigan, Ann Arbor, Michigan, United States of America; The Centre for Research and Technology, Hellas, Greece

## Abstract

The ortholog conjecture posits that orthologous genes are functionally more similar than paralogous genes. This conjecture is a cornerstone of phylogenomics and is used daily by both computational and experimental biologists in predicting, interpreting, and understanding gene functions. A recent study, however, challenged the ortholog conjecture on the basis of experimentally derived Gene Ontology (GO) annotations and microarray gene expression data in human and mouse. It instead proposed that the functional similarity of homologous genes is primarily determined by the cellular context in which the genes act, explaining why a greater functional similarity of (within-species) paralogs than (between-species) orthologs was observed. Here we show that GO-based functional similarity between human and mouse orthologs, relative to that between paralogs, has been increasing in the last five years. Further, compared with paralogs, orthologs are less likely to be included in the same study, causing an underestimation in their functional similarity. A close examination of functional studies of homologs with identical protein sequences reveals experimental biases, annotation errors, and homology-based functional inferences that are labeled in GO as experimental. These problems and the temporary nature of the GO-based finding make the current GO inappropriate for testing the ortholog conjecture. RNA sequencing (RNA-Seq) is known to be superior to microarray for comparing the expressions of different genes or in different species. Our analysis of a large RNA-Seq dataset of multiple tissues from eight mammals and the chicken shows that the expression similarity between orthologs is significantly higher than that between within-species paralogs, supporting the ortholog conjecture and refuting the cellular context hypothesis for gene expression. We conclude that the ortholog conjecture remains largely valid to the extent that it has been tested, but further scrutiny using more and better functional data is needed.

## Author Summary

Today's exceedingly high speed of genome sequencing, compared with the generally slow pace of functional assay, means that the functions of most genes identified from genome sequences will be annotated only through computational prediction. The primary source of information for this prediction is the functions of orthologous genes in model organisms, because orthologs are widely believed to be functionally similar, especially when compared with paralogs. This belief, known as the ortholog conjecture, was recently challenged on the basis of experimentally derived Gene Ontology (GO) annotations and microarray gene expression data, because these data revealed greater functional and expressional similarities of paralogs than orthologs. Here we show that GO-based estimates of functional similarities are temporary and unreliable, due to experimental biases, annotation errors, and homology-based functional inferences that are incorrectly labeled as experimental in GO. RNA sequencing (RNA-Seq) is superior to microarray for comparing the expressions of different genes or in different species, and our analysis of a large RNA-Seq dataset provides strong support to the ortholog conjecture for gene expression. We conclude that the ortholog conjecture remains largely valid to the extent that it has been tested, but further scrutiny using more and better functional data is needed.

## Introduction

Orthologs, or orthologous genes, are genes in different species that originated by vertical descent from a single gene of the last common ancestor [1]. By contrast, paralogs, or paralogous genes, are homologous genes separated by a gene duplication event [1]. They are referred to as inparalogs when the gene duplication postdated a particular speciation event of reference [2]. Otherwise, they are known as outparalogs [2]. Paralogs residing in the same species are called within-species paralogs, whereas those residing in different species are between-species paralogs. It is widely believed that orthologs are functionally more similar than paralogs, especially after the control of protein sequence dissimilarity or divergence time between genes [3]. This belief, formally termed the “ortholog conjecture” [3]–[4], is commonly used by molecular biologists in designing experiments and interpreting data and by computational biologists in predicting gene functions and annotating genome sequences [3], [5]–[9].

The theoretical basis of the “ortholog conjecture” is the consideration that, without duplication, a gene is unlikely to change its basic function because such a change would require the loss of the original function, which is usually harmful. Indeed, a recent evolutionary study of protein interaction suggests that the molecular function of a gene, in the absence of duplication, is highly conserved [10], although the biological processes in which the gene participates [11] and the importance of the gene [12]–[14] may be less conserved. With duplication, however, one gene copy may retain the original function, while the second copy can acquire new functions (i.e., neofunctionalization), resulting in functional divergence between the paralogs [15]. Alternatively, the two paralogs may each inherit some but not all of the progenitor gene's functions such that they together are functionally equivalent to the progenitor gene [16]. This process of subfunctionalization also leads to functional divergence between paralogs. Nonetheless, not all paralogs are expected to diverge in function. If an increased amount of gene product conferred by gene duplication is beneficial, the paralogs are expected to maintain their functions unaltered [17]. Additionally, subfunctionalization may occur with respect to the amount of gene expression, resulting in the total expression level of the two paralogs equivalent to that of the progenitor gene, which would also prevent the paralogs from functional divergence [18]. Overall, it seems likely that paralogs will diverge more rapidly than orthologs in gene function.

Although many genes from genetic model organisms have been extensively characterized functionally, the ortholog conjecture had never been systematically tested [19] until recently [4]. In a provocative paper, Nehrt and colleagues used experiment-based annotations in the Gene Ontology (GO) database [20] and microarray gene expression data [21] to compare the functional and expression similarities of orthologs and paralogs in human and mouse [4]. They showed that, given the same level of protein sequence divergence, (i) orthologs are less similar than paralogs and (ii) between-species paralogs are less similar than within-species paralogs, in function and expression [4]. They further showed that (iii) functional and expression similarities between orthologs are independent of the protein sequence identity between the orthologs. These results are inconsistent or contradictory to the ortholog conjecture, prompting the authors to propose that the primary determinant of the evolutionary rate of gene function and expression is the cellular context in which the genes act. This cellular context hypothesis could explain why within-species paralogs were observed to be more similar in function and expression than between-species paralogs and orthologs, when the degree of protein sequence divergence is controlled.

If the ortholog conjecture is indeed incorrect as claimed by Nehrt et al. [4], some fundamental models of molecular evolution and numerous computational predictions of gene functions would require major revisions. We, however, have doubts about the suitability of GO annotations for testing the ortholog conjecture, for several reasons. First, because of the wide belief of the ortholog conjecture, functional differences between orthologs may be perceived as more surprising than those between paralogs and may thus be preferentially published. Second, functional data of genes from different species tend to be annotated by different teams which may adopt different rules of annotation, which would artificially increase the functional dissimilarity of orthologs and between-species paralogs, compared with within-species paralogs. These and other potential biases in reporting and annotation may affect the test of the ortholog conjecture [22]–[23]. Nehrt et al. were aware of some potential biases in GO annotations. They thus also used microarray gene expression data from human and mouse to compare expression similarities between orthologs and between paralogs. But, microarray was primarily designed to compare the expressions of the same gene from the same species across conditions or tissues. As shown previously, comparison of microarray expression data of different genes or different species can be misleading, because of different microarray probes, designs, and normalizations [24]–[25]. Given the fundamental importance of the ortholog conjecture in biology and the above concerns, Nehrt et al's results require further scrutiny. Here we report biases and errors in GO annotations that prevent a fair evaluation of the ortholog conjecture. By contrast, the ortholog conjecture is strongly supported by RNA-Seq gene expression data, which are known to be superior to microarray data, especially for comparisons among different genes or species [24], [26]–[28]. The RNA-Seq data also reject the cellular context hypothesis for gene expression.

## Results

### Functional similarities of orthologs and paralogs in different versions of GO

The rapid accumulation of gene function data means that the number of annotations in GO has increased quickly in recent years [29]. It is interesting to examine whether functional similarities of orthologs and paralogs calculated based on GO annotations have remained relatively stable over time. Such stability is a necessary, albeit not sufficient, condition for drawing any meaningful conclusion from GO. Based on human and mouse GO releases from 2006 to 2011, we estimated the experiment-based functional similarities of orthologs and paralogs, using Nehrt et al.'s method [4]. Briefly, the functional similarity of a pair of homologous genes is the fraction of common experimentally derived functional annotations of the two genes (see Materials and Methods). To ensure comparability over years, we used the same set of genes for all years. That is, for each orthologous or paralogous gene pair, we estimated their functional similarity in 2006. We then calculated their functional similarity in each subsequent year, relative to that in 2006. By averaging across orthologs or paralogs, we measured the average functional similarity of orthologs or paralogs in each year, relative to that in 2006. Following Nehrt et al. [4], we examined three sets of gene pairs: (i) human-mouse orthologs, (ii) human and mouse within-species outparalogs, which were generated prior to the human-mouse separation, and (iii) human and mouse (within-species) inparalogs, which were generated after the human-mouse separation. The ortholog conjecture predicts a higher functional similarity between ortholog than the two types of paralogs. By contrast, Nehrt et al.'s cellular context hypothesis predicts a lower functional similarity between orthologs than the two types of within-species paralogs. We did not examine between-species outparalogs, because both hypotheses predict them to have relatively low functional similarities. For convenience, we refer to within-species outparalogs simply as outparalogs.

**Figure 1:**
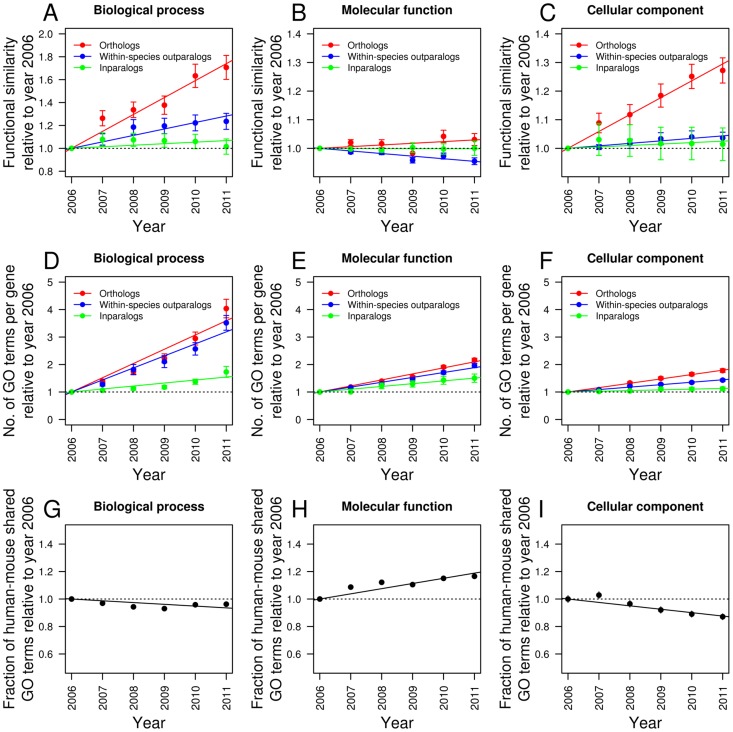
GO-based functional similarities of orthologs and paralogs vary in the last five years. (**A**–**C**) Functional similarities estimated from the GO annotations of different years, relative to those in 2006. In each of (A)–(C), the slopes of the three regression lines are significantly different from one another (*P*<0.02, two-tail *Z*-test), with the exception of the comparison between outparalogs and inparalogs in (C), which has a *P*-value of 0.08. (**D**–**F**) Numbers of GO annotations per gene in different years, relative to those in 2006. In each of (D)–(F), the slopes of the three regression lines are significant (*P*<0.002, two-tail *t*-test) and are significantly different from one another (*P*<0.0003, two-tail *Z*-test), with the exception of the comparison between outparalogs and orthologs in (D), which has a *P*-value of 0.09. (**G**–**I**) Fractions of shared GO terms between human and mouse in different years, relative to year 2006. The fraction is calculated by the number of overlapping GO terms divided by the total number of unique GO terms in human and mouse GO annotations. In each of (G)–(I), the slopes of the three regression lines are significantly different from 0 (*P*<0.012, two-tail *t*-test). In all panels, error bars indicate one standard error, although in some panels they are barely visible.

GO annotations are organized into three aspects: biological process, molecular function, and cellular component. Based on biological process GOs, the average functional similarity increased from year 2006 to 2011 for all three gene sets, but the increase was significantly faster for orthologs than the two types of paralogs (Fig. 1A; Table S1). The average annual increase in functional similarity is 14.8%, 5.6%, and 1.4% for orthologs, outparalogs, and inparalogs, respectively (*P*<10^−4^, *P*<10^−3^, and *P* = 0.073, respectively, *n* = 5, two-tail *t*-test) [30], and these annual increases are all significantly different from one another (*P*<10^−6^, two-tail *Z*-test) [31]. Thus, relative to paralogs, orthologs have become more similar in GO-annotated biological process functions over the last five years. We confirmed that this difference is not due to the difference in sample size between orthologs, outparalogs, and inparalogs (Fig. S1).

Based on molecular function GOs (Fig. 1B), functional similarity increased for orthologs (0.6% per year, *P* = 0.068, *n* = 5, two-tail *t*-test) but decreased for outparalogs (−0.9% per year, *P*<10^−3^, *n* = 5, two-tail *t*-test) and remained unchanged for inparalogs (−0.03% per year, *P* = 0.566, *n* = 5, two-tail *t*-test). Although the magnitudes of these changes are all small, the differences in annual change between orthologs and the two types of paralogs are both statistically significant (*P*<10^−7^ and 0.02, respectively, two-tail *Z*-test).

Based on cellular component GOs (Fig. 1C), the annual increase in functional similarity is 5.9%, 0.9%, and 0.5% for orthologs, outparalogs, and inparalogs, respectively (*P*<10^−5^, *P*<10^−4^, and *P* = 0.053, respectively, *n* = 5, two-tail *t*-test). The annual increases are significantly different between orthologs and the two types of paralogs (*P*<10^−6^, two-tail *Z*-test), but are not significantly different between inparalogs and outparalogs (*P* = 0.08, two-tail *Z*-test).

Thus, relative to the functional similarity between paralogs, functional similarity between orthologs has been increasing over the last five years in each of the three GO aspects, and there is no apparent deceleration of this increase (Fig. 1A–C). Although the absolute functional similarities of orthologs (0.40, 0.65, and 0.60 for biological process, molecular function, and cellular component, respectively) are still lower than those of outparalogs (0.55, 0.75, and 0.69) and inparalogs (0.67, 0.85, 0.79) in the latest GO version analyzed (Nov. 2011), these relations may be reversed in the future if the trend in Fig. 1A–C continues. Specifically, we predict based on the trend in Fig. 1A–C that functional similarity in biological process, molecular function, and cellular component would become greater for orthologs than outparalogs in 2013, 2018, and 2013, respectively. Similarly, functional similarity in the three GO aspects would become greater for orthologs than inparalogs in 2013, 2043, and 2015, respectively. These findings suggest that the current GO annotations do not allow a definitive conclusion about the ortholog conjecture.

What might have caused the differential rates of change in functional similarity between orthologs and paralogs over the last five years? Because of the wide acceptance of the ortholog conjecture, similar functions between orthologs may have been deemed uninteresting and hence underreported, especially for those orthologs with high sequence similarities. Nonetheless, as more and more gene function data from each species accumulate, the impact of such biases should decline, resulting in a relative increase in the functional similarity of orthologs over time. Alternatively, the patterns in Fig. 1A–C could be due to a slower increase in the number of GO annotations for orthologs than paralogs, because this number is the denominator in the definition of functional similarity [4] (see Materials and Methods). But, this potential explanation is incorrect. The numbers of GO annotations for the three types of homologs increased for each of the three aspects of GO (*P*<0.002, *n* = 5, two-tail *t*-test, Fig. 1D–F), and the increase in the number of GO annotations for orthologs is faster than those for outparalogs and inparalogs for each of the three aspects of GO (*P*<10^−6^, two-tail *Z*-test), with the exception of the comparison in biological process GOs between orthologs and outparalogs (*P* = 0.09, two-tail *Z*-test).

Another potential explanation is that there may be fewer organism-specific GO terms in later versions, which would boost the functional similarity between a randomly picked human gene and a randomly picked mouse gene as well as that between human-mouse orthologs. This possibility, however, can be ruled out for biological process GOs (Fig. 1G) and cellular component GOs (Fig. 1H), because the percentage of shared GO terms in human and mouse decreased by 1.3% (*P* = 0.012, *n* = 5, two-tail *t*-test) and 2.5% (*P*<0.001, *n* = 5, two-tail *t*-test) per year between 2006 and 2011 for these two aspects of GO, respectively. For molecular function GOs, the percentage of shared GO terms increased by 3.8% annually (*P*<0.001, *n* = 5, two-tail *t*-test; Fig. 1I), which may account for the relative increase of the molecular function similarity of orthologs over the years (Fig. 1B). Overall, these analyses suggest that the rising functional similarity of orthologs, especially in biological processes and cellular components, is likely due to previous underreporting of shared functions of orthologs. In the remainder of the paper, we analyze the latest GO version (Nov. 2011) unless otherwise noted.

### Homologous gene pairs studied in the same papers

**Figure 2:**
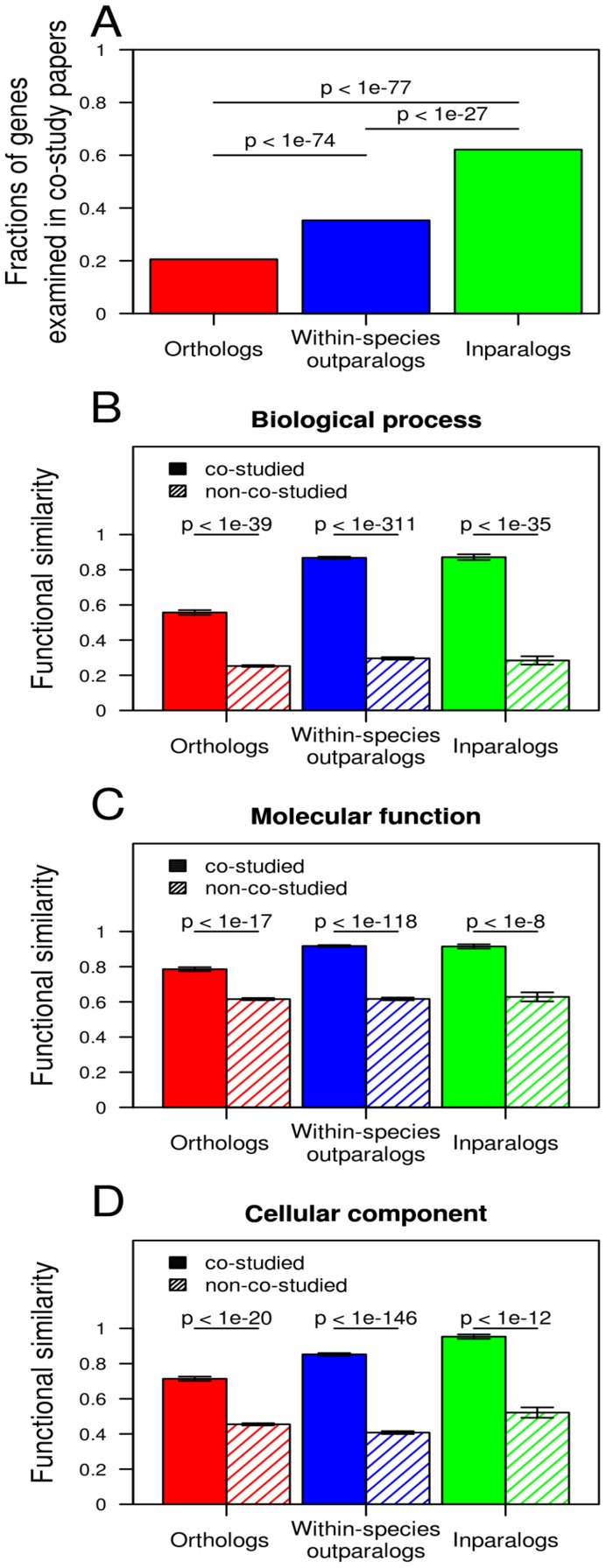
Biases in co-study papers that result in underestimation of functional similarity of ortholog, compared with paralogs. (**A**) Fractions of homologous gene pairs that have been co-examined in the same papers. *P* values are from a chi-square test. (**B**–**D**) Functional similarity of homologous genes estimated from co-study papers and other sources of data. Error bars indicate one standard error. *P* values are from *t*-tests.

Another potential bias in GO annotations is the source of functional data. We examined all papers used by GO that simultaneously studied a pair of homologous genes (co-study papers), and found that 21% of orthologs, 35% of outparalogs, and 62% of inparalogs in our dataset have been investigated in those co-study papers (Fig. 2A). Not unexpectedly, functional similarity between homologous genes appears higher in co-study papers than in other sources of information for both orthologs and paralogs in all three GO aspects (Fig. 2B–D). Thus, compared with paralogs, the under-representation of orthologs in co-study papers causes their functional similarity to appear lower.

To examine whether the temporal changes of functional similarity shown in Fig. 1 are primarily caused by the biases created by the co-study papers, we repeated the analysis after removing all co-study papers. We found the results to be qualitatively unaltered (Fig. S2), suggesting that the co-study papers cannot account for the temporal patterns of functional similarity in Fig. 1.

### Functional similarity of homologs with identical protein sequences

An unexpected observation in Fig. 2B–D is that, while the functional similarity is approximately equal among orthologs, outparalogs, and inparalogs when functional data outside co-study papers are used, the functional similarity is much greater for paralogs than orthologs in co-study papers. We wonder whether GO annotations based on co-study papers have underestimated the functional similarity of orthologs, compared with paralogs. To address this question, we investigated among the co-study papers those that studied homologs with identical protein sequences, because these gene pairs should have highly similar if not identical functions that are dependent on protein sequences (Table S2). These homologs include 31 orthologous pairs, five inparalogous pairs, and four outparalogous pairs. All nine paralogous pairs have 100% functional similarity in the GO annotations based on the co-study papers, while this occurs to only nine of the 31 orthologous pairs (*P* = 0.0002, Fisher's exact test). More extremely, eight of the 31 orthologous pairs show 0% functional similarity (Table 1). Surprisingly, none of the co-study papers [32]–[39] of these eight orthologous pairs explicitly mentioned functional dissimilarity between these orthologs. Several biases and errors are apparent. First, many so-called experiment-based GO annotations are inferred based on the experiments of a homolog of the gene being annotated. For example, the molecular function of “protein binding” for human gene encoding GABA(A) receptor-associated protein (ENSG00000170296) was inferred from an experiment with a monkey homolog rather than the human gene itself (first case in Table 1). Similarly, the three cellular component annotations for the mouse ortholog of the human gene were inferred from rat (first case in Table 1). Such between-species functional inferences in so-called experiment-based GO annotations make the test of the ortholog conjecture circular. Second, different experiments were often conducted for two orthologs in the same co-study paper, probably because many experiments are not equally feasible in two species. This practice necessarily renders the estimated functional similarity of orthologs low. The second case in Table 1 illustrates this point, where the human ortholog was examined for molecular function while the mouse ortholog was examined for cellular component. Third, annotation errors are also prevalent. For example, GO annotated human interleukin enhancer binding factor 2 (ILF2, ENSG00000143621) as having a molecular function of “DNA binding” based on the paper with a PMID of 11804788 (third case in Table 1), but nowhere in this paper was this molecular function experimentally demonstrated. Sometimes, an experiment was conducted in one species but annotated for another species. For instance, the mouse but not the human gene encoding ras-related C3 botulinum toxin substrate 3 (Rac3, ENSG00000169750) was annotated for the biological process of “neuron projection development”, despite that the experiment was done in human cells (fourth case in Table 1). These homology-based functional inferences, experimental biases, and annotation errors, together with the biases identified earlier and the temporariness of the GO-based finding, suggest that the ortholog conjecture cannot be tested with the current GO annotations.

**Table 1:** Eight pairs of human-mouse orthologs with identical protein sequences but no overlapping GO annotations based on co-study papers.

PubMed ID	Ensembl gene ID	GO term	GO category	GO term description	Experimental systems^1^	Bias/error^2^
PMID:11146101	ENSG00000170296	GO:0005515	Molecular function	protein binding	Monkey	Inferred function
	ENSMUSG00000018567	GO:0005790	Cellular component	smooth endoplasmic reticulum	Rat	Experimental bias
		GO:0005794	Cellular component	Golgi apparatus	Rat	
		GO:0005764	Cellular component	lysosome	Rat	
PMID:11302691	ENSG00000215021	GO:0005515	Molecular function	protein binding	Human	Experimental bias
	ENSMUSG00000004264	GO:0005743	Cellular component	mitochondrial inner membrane	Mouse	
PMID:11804788	ENSG00000143621	GO:0003677	Molecular function	DNA binding	–	Annotation error
	ENSMUSG00000001016	GO:0005634	Cellular component	nucleus	Mouse	Experimental bias
		GO:0005730	Cellular component	nucleolus	Mouse	
PMID:16525025	ENSG00000169750	GO:0030426	Cellular component	growth cone	Rat, human	Annotation error
		GO:0043025	Cellular component	neuronal cell body	Rat, human	Inferred function
		GO:0043005	Cellular component	neuron projection	Rat, human	
		GO:0031941	Cellular component	filamentous actin	Rat, human	
	ENSMUSG00000018012	GO:0031175	Biological process	neuron projection development	Rat, human	
PMID:11595183	ENSG00000155849	GO:0005886	Cellular component	plasma membrane	Hamster	Annotation error
		GO:0005737	Cellular component	cytoplasm	Hamster	Experimental bias
		GO:0016601	Biological process	Rac protein signal transduction	Hamster	Inferred function
		GO:0006928	Biological process	cellular component movement	Human, hamster	
		GO:0006911	Biological process	phagocytosis, engulfment	Human, hamster	
		GO:0030036	Biological process	actin cytoskeleton organization	Hamster	
	ENSMUSG00000041112	GO:0030029	Biological process	actin filament-based process	Hamster	
		GO:0006909	Biological process	phagocytosis	Hamster	
PMID:15004007	ENSG00000087095	GO:0007179	Biological process	transforming growth factor beta receptor signaling pathway	Human (frog gene)	Annotation error
	ENSMUSG00000017376	GO:0033136	Biological process	serine phosphorylation of STAT3 protein	Human (frog gene)	Experimental bias
		GO:0004674	Molecular function	protein serine/threonine kinase activity	Human (frog gene)	Inferred function
		GO:0042169	Molecular function	SH2 domain binding	Human (frog gene)	
PMID:18339854	ENSG00000119048	GO:0004842	Molecular function	ubiquitin-protein ligase activity	Human	Experimental bias
		GO:0005737	Cellular component	cytoplasm	Human, mouse	Annotation error
		GO:0005634	Cellular component	nucleus	Human, mouse	
		GO:0006513	Biological process	protein monoubiquitination	Human	
		GO:0033522	Biological process	histone H2A ubiquitination	Human	
		GO:0050821	Biological process	protein stabilization	Human	
		GO:0000209	Biological process	protein polyubiquitination	Human	
	ENSMUSG00000020390	GO:0060070	Biological process	canonical Wnt receptor signaling pathway	Mouse	
PMID:19154719	ENSG00000198435	GO:0045746	Biological process	negative regulation of Notch signaling pathway	Human	Experimental bias
		GO:0090263	Biological process	positive regulation of canonical Wnt receptor signaling pathway	Human	
	ENSMUSG00000078202	GO:0002043	Biological process	blood vessel endothelial cell proliferation involved in sprouting angiogenesis	Mouse	
		GO:0001569	Biological process	patterning of blood vessels	Mouse	
		GO:0001938	Biological process	positive regulation of endothelial cell proliferation	Mouse	
		GO:0022407	Biological process	regulation of cell-cell adhesion	Mouse	
		GO:0002040	Biological process	sprouting angiogenesis	Mouse	

1 Experimental systems may mean organisms or cell lines.

2 Bias/error indicates (1) experimental bias (i.e., different functional aspects were examined for orthologs), (2) inferred function (i.e., function in a species is inferred from that in a related species), or (3) annotation error (i.e., mistake in annotation).

### RNA-Seq expression similarities between orthologs and between paralogs

Because the biases and errors in GO are hard to control, we, like Nehrt et al. [4], turned to genome-wide gene expression data, which are not subject to the type of biases in GO, because they were systematically generated at the genomic scale. While gene expression does not equal gene function, the expression level and pattern of a gene must be more or less concordant with its function such that expression similarity between genes should reflect their functional similarity to some degree [40]. The problems of using microarray data to measure expression similarities between genes and species have been well documented [24]–[25]. For instance, without appropriate normalization, an earlier study of microarray data reported the unexpected finding that the gene expression patterns of two different tissues from the same species (e.g., human heart and human testis) are more similar than those from the same tissue of different species (e.g., human heart and mouse heart) [41]. After the control of differential hybridizations between orthologs caused by probe differences, the above relation is reversed [25]. By contrast, RNA-Seq is immune to the probe bias [26] and has correctly revealed the lower expression similarity between different tissues of the same species than the same tissue of different species [42]–[43]. The dynamic range of RNA-Seq is also much greater than microarray and the linear relationship between cDNA concentrations and expression estimates is better in RNA-Seq than microarray, making RNA-Seq a preferred method of expression quantification [24], [26]. Here we use a recently published RNA-Seq dataset that includes six male and four female tissues of human and mouse [42] to test the ortholog conjecture.

**Figure 3:**
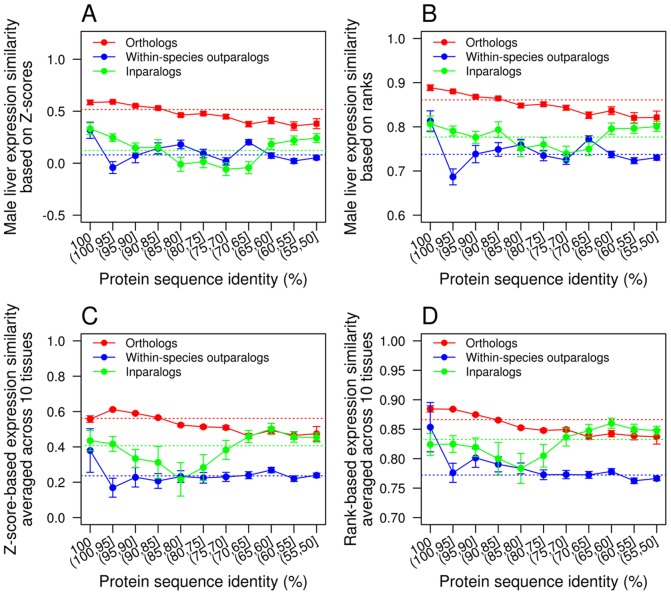
Expression similarity of homologous genes. (**A**) Male liver expression similarity based on expression *Z*-scores. (**B**) Male liver expression similarity based on expression ranks. (**C**) Mean expression similarity of 10 tissues based on expression *Z*-scores. (**D**) Mean expression similarity of 10 tissues based on expression ranks. Error bars show one standard error. Each solid line connects the expression similarity values of different bins, whereas the dotted line shows the mean value across all genes in all bins. In each panel, the red dotted line is significantly higher than the green line (*P*<10^−10^, two-tail *Z*-test), which is in turn significantly higher than the blue line (*P*<10^−10^, two-tail *Z*-test).

In the RNA-Seq data, gene expression is measured in RPKM, standing for reads per kilobase of exon model per million mapped reads [44]. Because the distributions of gene expression levels differ substantially between human and mouse, it is inappropriate to compare the expression levels of human and mouse orthologs directly [18]. We thus transformed the expression levels of human and mouse genes to *Z*-scores after a log_2_ transformation of RPKM values (see Materials and Methods) [18]. That is, we draw a distribution of log_2_(RPKM) for all genes in the genome and calculate the mean and standard deviation of the distribution; the *Z*-score of a gene is the distance between its log_2_(RPKM) value and the mean of the distribution, divided by the standard deviations of the distribution. The expression similarity between homologs is a measure of similarity in *Z*-score between the two genes (see Materials and Methods). We analyzed each tissue separately, because recent studies have shown that comparing across-tissue expression-profile similarities of different gene pairs using either Pearson's correlation or Euclidian distances is problematic, because the variation of tissue-specificity of expression among genes interferes with such a comparison [45]–[46]. We first describe the observations in the male liver. We found the expression similarity between orthologs significantly higher than that between inparalogs and that between outparalogs, with or without the control of protein sequence identity (Fig. 3A; Table S3). Further, expression similarity of orthologs declines with the decrease of protein sequence identity (*n* = 11, Pearson's correlation coefficient *r* = 0.96, *P*<10^−5^), suggesting that gene expression evolution and protein sequence evolution are correlated [25], [47]. Using expression ranks (see Materials and Methods) instead of *Z*-scores yielded the similar result of higher expression similarities between orthologs than between inparalogs and between outparalogs (Fig. 3B). Similarly, rank-based expression similarity of orthologs declines with the decrease of sequence identity (*n* = 11; *r* = 0.97, *P*<10^−6^). All nine other tissues examined show generally similar patterns, except that in a number of tissues (e.g., testis) inparalogs become more similar than orthologs when the protein sequence identity is low (Figs. S3 and S4). This is probably an artifact caused by the low expression levels of the inparalogs with low protein sequence identities (Fig. S5). By definition, these inparalogs evolve rapidly in protein sequence. They tend to be lowly expressed, because of the strong negative correlation between protein expression levels and evolutionary rates [48]–[51]. If two genes are both lowly expressed, their expression divergence would tend to be small. This bias is less severe for orthologs and outparalogs with similar levels sequence identity because they have longer divergence times and thus lower evolutionary rates than the inparalogs. Consequently, their expression levels are not so low (Fig. S5) and their expression similarities are not so high. We also estimated the average expression similarity across all 10 tissues by *Z*-scores (Fig. 3C; Table S3) or expression ranks (Fig. 3D). The results are similar to those from individual tissues.

**Figure 4:**
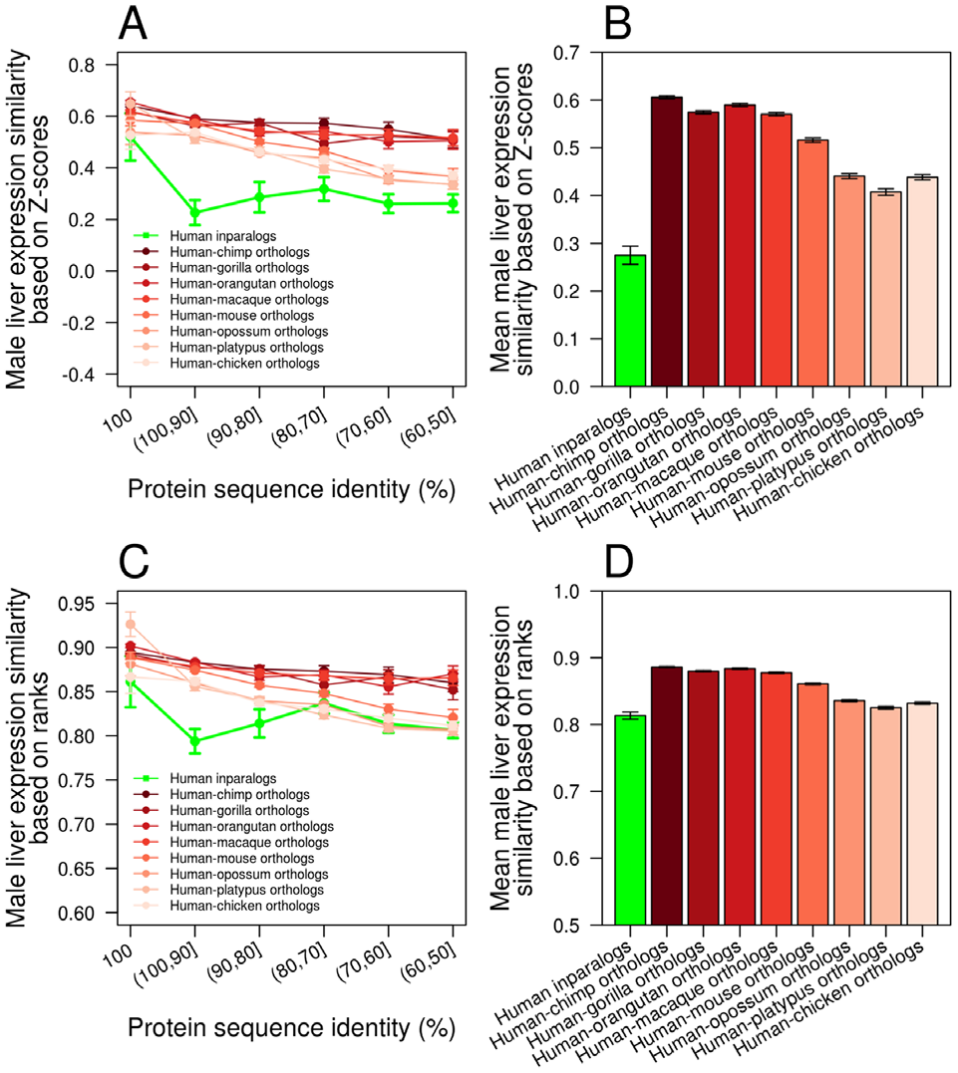
Male liver expression similarity of homologous genes from multiple species. (**A**) *Z*-score-based expression similarities of human inparalogs are lower than those of orthologs between human and multiple species, for all protein sequence identity bins. (**B**) Average *Z*-score-based expression similarity of human inparalogs and those of orthologs between human and multiple species. The green bar is significantly lower than each of the red bars (*P*<10^−10^, two-tail *Z*-test). (**C**) Rank-based expression similarities of human inparalogs are lower than those of orthologs between human and multiple species, for most protein sequence identity bins. (**D**) Average rank-based expression similarity of human inparalogs and those of orthologs between human and multiple species. The green bar is significantly lower than each of the red bars (*P*<0.04, two-tail *Z*-test). For all panels, error bars indicate one standard error.

In addition to measuring the functional similarity of orthologs between human and mouse, we also measured it between human and all other species in the RNA-Seq data, including chimpanzee, gorilla, orangutan, macaque, opossum, platypus, and chicken [42]. We found that the *Z*-score-based expression similarity of human inparalogs generated after the human-mouse separation is lower than the expression similarity of orthologs between human and all above species, with (Fig. 4A; Table S4) or without (Fig. 4B) the control of protein sequence identity, when the male liver is examined. We also found the mean expression similarity of orthologs correlated with the divergence time of orthologs (*n* = 8, Spearman's correlation coefficient ρ = −0.95, *P* = 0.001; Fig. 4B). Similar results were observed when rank-based expression similarities were used (Fig. 4C, D). These patterns are generally common among all tissues examined (Figs. S6 and S7). Nehrt et al.'s cellular context hypothesis [4] asserts that the divergence of cellular context in which genes act is the key determinant of the functional divergence of homologous genes. It predicts that functional similarity between orthologs is lower than that between within-species paralogs, regardless of the divergence time between the orthologs and that between the paralogs. Hence, the above results (Fig. 4; Figs. S6 and S7) are inconsistent with the cellular context hypothesis.

**Figure 5:**
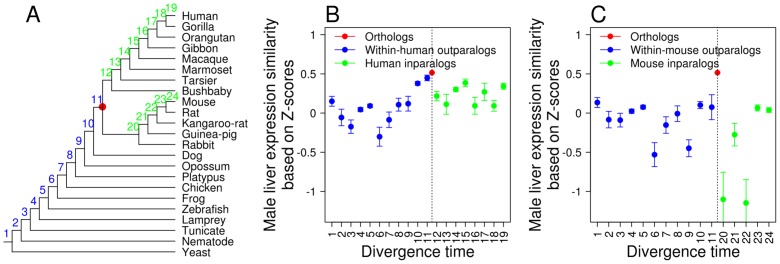
*Z*-score-based male liver expressions are more similar between human-mouse orthologs than between within-species paralogs. (**A**) A species phylogeny with numbered branches in which various paralogs were generated. The red dot indicates the time of human-mouse separation. (**B**) *Z*-score-based male liver expression similarity for human-mouse orthologs and within-human paralogs of different ages. Numbers on the X-axis correspond to the branches in (A). Error bars indicate one standard error. The dashed line indicates the human-mouse divergence. (**C**) *Z*-score-based male liver expression similarity for human-mouse orthologs and within-mouse paralogs of different ages.

Using male liver expression similarities of homologous genes estimated from the RNA-Seq data, we further tested Nehrt et al.'s cellular context hypothesis. Contrary to its prediction, we found the *Z*-score-based expression similarity between human-mouse orthologs always higher than that between within-human paralogs (*P*≤0.031, one-tail *Z*-test) or within-mouse paralogs (*P*≤0.003, one-tail *Z*-test), irrespective of the divergence time of the paralogs (Fig. 5). This pattern is also generally observed in nine other tissues examined (Fig. S8). Use of rank-based expression similarities yielded similar results (Fig. S9). Together, the RNA-Seq data support the ortholog conjecture and refute the cellular context hypothesis.

## Discussion

Stimulated by Nehrt et al.'s pioneering test of the ortholog conjecture with large-scale functional and expression data [4], we here examined the suitability of such data for testing this conjecture. We found several biases and errors in the current GO that limit its utility. First, we observed a steady increase over the last five years of the functional similarity between orthologs, compared with that between paralogs. Hence, even if today's GO shows a lower functional similarity between orthologs than between paralogs, this relation may be reversed in the future when there are more GO annotations. Second, compared with paralogs, orthologs are underrepresented in co-study papers that simultaneously examined homologous genes. Because co-study papers tend to report higher functional similarities than other papers, functional similarity of orthologs is underestimated, relative to that of paralogs. This result is consistent with a recent analysis of GO [52]. Third, a close examination of 31 co-study papers that studied homologs with identical protein sequences revealed that orthologs are more likely than paralogs to be subjected to different experiments, causing underestimation of functional similarity of orthologs. The high prevalence of this experimental bias observed from our relatively small sample is consistent with the recent report of the GO Consortium [23]. Fourth, the above examinations also revealed GO annotation errors that reduce the functional similarity of orthologs. Fifth and most disturbingly, the so-called experiment-based functional evidence in GO often originates from species other than the one being annotated. In other words, such functional data were inferred from those of homologous genes, rendering the entire test of the ortholog conjecture by GO circular. Because spotting such problems requires careful reading of original papers, we do not know the prevalence of this problem in GO at large. But four of the eight orthologous gene pairs in Table 1 involve such homology-based functional inferences.

Very recently, Altenhoff et al. also reported a number of biases in GO annotations [52]. In addition to the co-study bias discussed above, they also found variation of GO term frequency among species, variation of background similarity among species pairs, and propagated annotation bias. These authors suggested that functional similarity between orthologs is slightly but significantly higher than that between paralogs, when these four biases are controlled. We are unsure about the reliability of their conclusion, because of the additional biases we detected that were not controlled in their study. Of special concern is the “contamination” of so-called experiment-based functional data by those inferred from homologs. The steady increase of the GO functional similarity between orthologs, relative to that between paralogs, over the last five years means that Altenhoff et al.'s result is at best temporary. With these considerations, we believe that the current GO cannot serve as a solid base for any conclusion regarding the ortholog conjecture. But we are aware of the ongoing improvement of GO annotations [29] and hope that GO will become useful for resolving the ortholog conjecture and other important biological problems in the future.

Our analysis of RNA-Seq gene expression data, which are more suitable than microarray data for comparing expression levels of different genes and different species, provides strong evidence for the ortholog conjecture. We observed that expression similarity between orthologs is generally higher than that between paralogs, with or without controlling the protein sequence identity or divergence time between genes. We believe that Nehrt et al.'s finding of a lower expression similarity of orthologs than that of within-species paralogs was likely caused by the incomparability between different microarrays that artificially reduces between-species expression similarities [25]. This bias may be alleviated when orthologs and between-species paralogs are compared due to the occurrence of the same bias to both types of gene pairs. Indeed, when orthologs and between-species outparalogs are compared, both Nehrt et al. [4] and another recent study [53] found orthologs to be more similar in expression than outparalogs, consistent with the ortholog conjecture. Our RNA-Seq results also refute the cellular context hypothesis proposed by Nehrt et al. [4], because we found orthologs to be more similar in expression than within-species paralogs (Figs. 4 and 5), opposite to the prediction of this hypothesis.

We note that the definition of the ortholog conjecture is vague in that it does not clearly state whether (i) any orthologs and paralogs, (ii) only those with the same divergence time, or (iii) only those with the same sequence identity can be compared. Nehrt et al. [4] compared orthologs and paralogs of the same protein sequence identity. In our analysis of the gene expression data, all three comparisons were made. For example, Fig. 3 used (i) and (iii). Fig. 4 used (ii) to show that inparalogs are functionally less similar than orthologs of various divergence times, including those that are older than the inparalogs. Fig. 5 also used (ii) to show that orthologs are functionally more similar than paralogs of similar divergence times or even smaller divergence times.

Although our genome-wide gene expression analysis supports the ortholog conjecture, we caution that this is not the final proof of the ortholog conjecture, because it should be further tested with large-scale gene function data. Even in terms of gene expression, the ortholog conjecture could be further tested with splicing variants and protein expression levels when such data in multiple species/tissues become available. It is notable that, in addition to Nehrt et al. [4], there are other lines of evidence from large-scale functional data that appear to be at odds with the ortholog conjecture [19], [54]. For example, based on experimental as well as predicted protein subcellular localization data, we previously showed that the rate of protein subcellular relocalization during fungal evolution is not lower for orthologs than paralogs [55], although a recent GO-based analysis suggests otherwise [52]. Another example is the report that protein interactions appear to be more similar between within-species paralogs than between orthologs [56], but this finding is likely an artifact arising from vastly different coverages of high-throughput protein interaction data of different species. In fact, a low-throughput targeted study found that protein interactions are highly conserved between orthologs [10], but a comparable study of paralogs is currently lacking. What is clear, however, is the complexity and difficulty of testing the ortholog conjecture with large, systematic, and reliable functional data. The biases and errors in GO identified here, coupled with those reported recently [23], [52], caution the interpretation of findings made from GO annotations. Despite its wide applications, GO information is registered and annotated by individual bioinformaticians based on studies designed and conducted by individual investigators. The shear size of the information in GO does not necessarily translate to quality or reliability. We conclude that to date there is no unambiguous genome-wide evidence against the ortholog conjecture, but further tests are needed.

## Materials and Methods

### Comparative genomic data

Human and mouse within-species paralogs, and one-to-one orthologs between human and chimpanzee, gorilla, orangutan, macaque, mouse, opossum, platypus, and chicken were downloaded from EnsEMBL (release 64, Sept. 2011). Protein sequence identity, information of the most recent common ancestor of a pair of paralogs, mapping of EnsEMBL Gene ID to UniProt/SwissProt Accession in human and MGI ID in mouse were also downloaded from EnsEMBL [57]. The information of the most recent common ancestor of within-species paralogs allows us to determine if the duplication occurred before (outparalogs) or after (inparalogs) the separation between human and mouse. In total, our dataset is composed of 20,799 human genes and 23,255 mouse genes, including 15,588 pairs of orthologs, 55,578 pairs of inparalogs, and 233,295 pairs of within-species outparalogs. The number of orthologs between human and chimpanzee, gorilla, orangutan, macaque, opossum, platypus, and chicken is 17,689, 15,692, 16758, 16,211, 13,475, 10,832, and 11,205, respectively.

### Gene Ontology (GO) annotations

GO annotations [58] in biological process, molecular function, and cellular component were retrieved from the GO database. We downloaded the lite version of GO-gene associations and used annotations with the evidence code of EXP (inferred from experiment) and its children (IDA, inferred from direct assay; IEP, inferred from expression pattern; IGI, inferred from genetic interaction; IMP, inferred from mutant phenotype; and IPI, inferred from physical interaction). Because GO is represented by directed acyclic graphs (DAGs), the original functional terms were propagated towards the root of each DAG (with the root node excluded), producing a complete set of terms for each gene. OBO v1.2 (version 12:10:2011) was used for GO term propagation. The “Is-A” type of relations from OBO was used to propagate GO terms.

We used the same version of OBO (12:10:2011) in the time series analysis (Fig. 1). Using OBO versions from individual years did not alter our results.

In total, 1,818 human and 3,515 mouse proteins had at least one propagated GO term in all GO annotation releases in the November of each year from 2006 to 2011. Homologous gene pairs with protein sequence identity greater than 50% were used for estimating functional similarity. The final number of homologous gene pairs in our GO time series assay was 1,077 orthologs, 105 inparalogs, and 1,027 within-species outparalogs. The significance of the annual increase in functional similarity was assessed by the significance of the regression coefficient in a *t*-test [30]. The difference in annual increase between two independent samples was evaluated by testing the equality of regression coefficients in a *Z*-test [31].

In the GO annotation release of November 2011, there were 4,494 orthologous pairs, 404 inparalogous pairs, and 13,449 within-species outparalogous pairs with associated PubMed IDs for both genes. Homologous pairs sharing at least one PubMed ID were identified as being studied in a co-study paper. If there is more than one co-study paper for a homologous pair, we chose the paper that annotated more shared GO terms for further analysis. In total, 924 orthologous pairs, 251 inparalogous pairs, and 4,751 within-species outparalogous pairs were co-studied.

### RNA-Seq data

The RNA-Seq-based gene expression levels were obtained from Brawand et al. [42], which included six male and four female tissues from humans and six male and five female tissues from mice. Six male tissues and four female tissues shared between human and mouse were used for further analysis. We calculated RPKM values of each gene from the Rz values in [42]. Rz is the mean per-base read coverage for each gene, computed for Ensembl-annotated exons with reads unambiguously mapped by TopHat (version 1.0.13) [42].

To make the expression levels comparable between species and tissues, we first calculated log_2_(RPKM) and then transformed it to a *Z*-score within each tissue of each species. As a result, gene expressions within a tissue of a species have a mean of 0 and a standard deviation of 1 [18]. To be included in subsequent analyses, both members of a homologous gene pair must have non-zero RPKM values. In addition to the *Z*-score-based analysis, we ranked genes in each tissue of each species according to their expression levels and converted the ranks to percentile ranks. In total, we analyzed the expression levels of 12,219 human genes and 12,048 mouse genes in all 10 tissues.

### Functional similarity and expression similarity

There are several different measures of functional similarity in the literature [59], but a recent study found them to yield similar results [52]. We used the measure proposed by Nehrt et al. [4] to calculate GO-based functional similarity between a pair of genes so that our results are directly comparable to theirs. Let *T_i_* be the set of propagated GO terms for gene *i* and *T_j_* be the corresponding set for gene *j*. Functional similarity between *i* and *j* is defined by 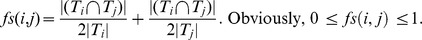



We used the expression *Z*-scores in each tissue to estimate expression similarity between genes. Let *Z*_i_ be a *Z*-score for gene *i* and *Z*_j_ be the *Z*-score for gene *j* in the tissue concerned. The expression similarity between *i* and *j* was calculated by 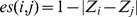, which has a maximal value of 1. We similarly estimated the expression similarly using percentile ranks, with the replacement of *Z*-scores by expression percentile ranks.

## Supporting Information

Figure S1 GO-based functional similarities of orthologs and paralogs of different years, relative to those in 2006, in (**A**) biological process, (**B**) molecular function, and (**C**) cellular component. This figure is identical to Fig. 1A–C, except that we randomly sample equal numbers of orthologs and outparalogs as that of inparalogs. The averages of 1000 replications of the random sampling are presented.
(PDF)

Figure S2 GO-based functional similarities of orthologs and paralogs of different years, relative to those in 2006, in (**A**) biological process, (**B**) molecular function, and (**C**) cellular component. This figure is identical to Fig. 1A–C, except that we excluded the annotations from co-study papers. Based on biological process GOs, the average annual increase in functional similarity is 6.9%, 4.2%, and −6.3% for orthologs, outparalogs, and inparalogs, respectively (*P* = 0.0001, 0.004, and 0.0004, respectively, *n* = 5, two-tail *t*-test), and these annual increases are all significantly different from one another (*P*<0.009, two-tail *Z*-test). Based on molecular function GOs, the average annual increase in functional similarity is 0.0%, −1.4%, and 1.5% for orthologs, outparalogs, and inparalogs, respectively (*P* = 0.9, 0.00007, and 0.02, respectively, *n* = 5, two-tail *t*-test), and these annual increases are all significantly different from one another (*P*<0.004, two-tail *Z*-test). Based on cellular component GOs, the average annual increase in functional similarity is 5.2%, 0.8%, and −0.8% for orthologs, outparalogs, and inparalogs, respectively (*P* = 0.00003, 0.004, and 0.0003, respectively, *n* = 5, two-tail *t*-test), and these annual increases are all significantly different from one another (*P*<10^−11^, two-tail *Z*-test).
(PDF)

Figure S3 Expression similarities of homologous genes in (A) male brain, (B) male cerebellum, (C) male heart, (D) male kidney, (E) male testis, (F) female brain, (G) female cerebellum, (H) female heart, and (I) female kidney, based on *Z*-scores. Error bars show one standard error. Each solid line connects the expression similarity values of different bins, whereas the dotted line shows the mean value across all genes in all bins.
(PDF)

Figure S4 Expression similarities of homologous genes in (A) male brain, (B) male cerebellum, (C) male heart, (D) male kidney, (E) male testis, (F) female brain, (G) female cerebellum, (H) female heart, and (I) female kidney, based on expression ranks. Error bars show one standard error. Each solid line connects the expression similarity values of different bins, whereas the dotted line shows the mean value across all genes in all bins. Note that in male testis the expression similarity between orthologs is almost identical to that between inparalogs (*P* = 0.49, one-tail *Z*-test).
(PDF)

Figure S5 Variations of mean expression levels of orthologs, outparalogs, and inparalogs with different protein sequence identities.
(PDF)

Figure S6 *Z*-score-based expression similarity of homologous genes from multiple species, examined in nine tissues. Expression similarities of human inparalogs (green dots) are generally lower than those of orthologs between human and multiple species (red dots) for individual protein sequence identity bins in each of the nine tissues: (A) male brain, (B) male cerebellum, (C) male heart, (D) male kidney, (E) male testis, (F) female brain, (G) female cerebellum, (H) female heart, and (I) female kidney. Mean expression similarity of human inparalogs (green bar) and those of orthologs between human and multiple species (red bars) in each of the nine tissues: (J) male brain, (K) male cerebellum, (L) male heart, (M) male kidney, (N) male testis, (O) female brain, (P) female cerebellum, (Q) female heart, and (R) female kidney. For all panels, error bars indicate one standard error. Expression data from certain tissues are not available in some species.
(PDF)

Figure S7 Rank-based expression similarity of homologous genes from multiple species, examined in nine tissues. Expression similarities of human inparalogs (green dots) are generally lower than those of orthologs between human and multiple species (red dots) for individual protein sequence identity bins in each of the nine tissues: (A) male brain, (B) male cerebellum, (C) male heart, (D) male kidney, (E) male testis, (F) female brain, (G) female cerebellum, (H) female heart, and (I) female kidney. Mean expression similarity of human inparalogs (green bar) and those of orthologs between human and multiple species (red bars) in each of the nine tissues: (J) male brain, (K) male cerebellum, (L) male heart, (M) male kidney, (N) male testis, (O) female brain, (P) female cerebellum, (Q) female heart, and (R) female kidney. For all panels, error bars indicate one standard error. Expression data from certain tissues are not available in some species.
(PDF)

Figure S8 *Z*-score-based expressions are generally more similar for human-mouse orthologs than (A-I) within-human paralogs and (J-R) within-mouse paralogs across nine tissues. Numbers on the X-axis correspond to the branches in Fig. 5A. Error bars indicate one standard error. The dashed line indicates the human-mouse divergence.
(PDF)

Figure S9 Rank-based expressions are generally more similar for human-mouse orthologs than (A-J) within-human paralogs and (K-T) within-mouse paralogs across 10 tissues. Numbers on the X-axis correspond to the branches in Fig. 5A. Error bars indicate one standard error. The dashed line indicates the human-mouse divergence.
(PDF)

Table S1 Numbers of gene pairs used in the time series analysis (Fig. 1 and Fig. S2). Note that the gene pairs used are the same across all years.
(DOC)

Table S2 Human and mouse homologous genes with identical protein sequences in co-study papers.
(DOC)

Table S3 Numbers of gene pairs used at each level (bin) of sequence identity in human and mouse RNA-Seq analysis (Fig. 3).
(DOC)

Table S4 Numbers of gene pairs used at each level (bin) of sequence identity in the multiple-species RNA-Seq analysis (Fig. 4).
(DOC)
