# Correction: The Ortholog Conjecture Is Untestable by the Current Gene Ontology but Is Supported by RNA Sequencing Data

**DOI:** 10.1371/annotation/6b5adbad-8944-4ab4-acd9-ac6f0d3e624e

**Published:** 2013-01-31

**Authors:** Xiaoshu Chen, Jianzhi Zhang

Figure S3 was inadvertently linked to Figure S4. Please view the correct Figure S3 here: 
