# Supplementary figures and images for: The Ortholog Conjecture Is Untestable by the Current Gene Ontology but Is Supported by RNA Sequencing Data
Source: PLoS Comput Biol. 2012 Nov 29;8(11):e1002784. doi: 10.1371/journal.pcbi.1002784 (PMC3510086; doi:10.1371/journal.pcbi.1002784)

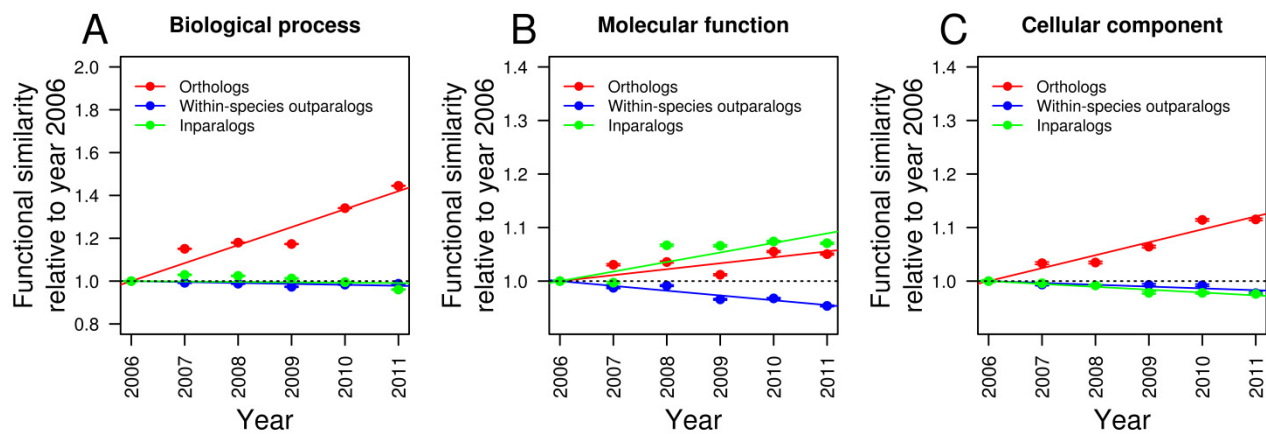

Figure S1

Supplement: Figure S1 — GO-based functional similarities of orthologs and paralogs of different years, relative to those in 2006, in (A) biological process, (B) molecular function, and (C) cellular component. This figure is identical to Fig. 1A–C, except that we randomly sample equal numbers of orthologs and outparalogs as that of inparalogs. The averages of 1000 replications of the random sampling are presented. (PDF) [file pcbi.1002784.s001.pdf]

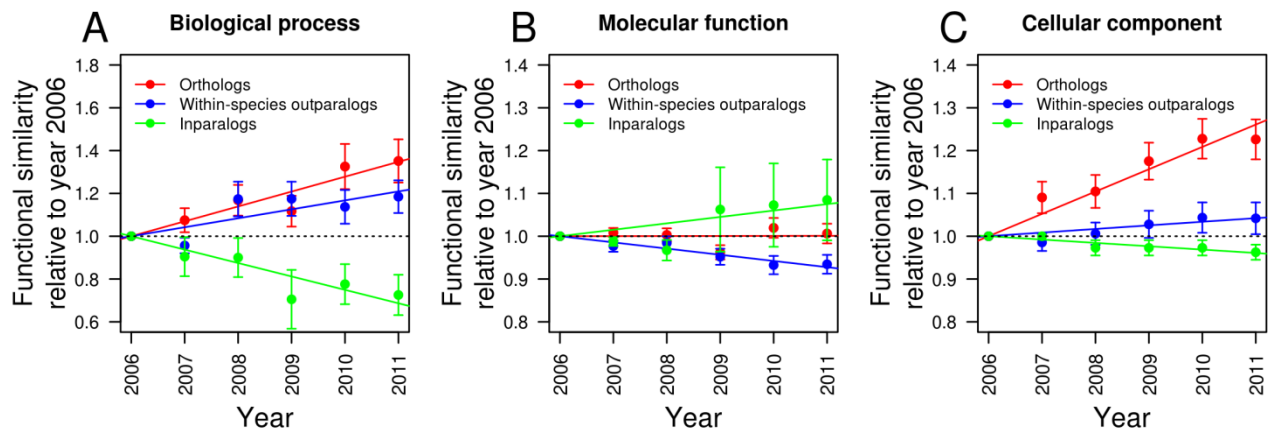

Figure S2

Supplement: Figure S2 — GO-based functional similarities of orthologs and paralogs of different years, relative to those in 2006, in (A) biological process, (B) molecular function, and (C) cellular component. This figure is identical to Fig. 1A–C, except that we excluded the annotations from co-study papers. Based on biological process GOs, the average annual increase in functional similarity is 6.9%, 4.2%, and −6.3% for orthologs, outparalogs, and inparalogs, respectively (P = 0.0001, 0.004, and 0.0004, respectively, n = 5, two-tail t-test), and these annual increases are all significantly different from one another (P<0.009, two-tail Z-test). Based on molecular function GOs, the average annual increase in functional similarity is 0.0%, −1.4%, and 1.5% for orthologs, outparalogs, and inparalogs, respectively (P = 0.9, 0.00007, and 0.02, respectively, n = 5, two-tail t-test), and these annual increases are all significantly different from one another (P<0.004, two-tail Z-test). Based on cellular component GOs, the average annual increase in functional similarity is 5.2%, 0.8%, and −0.8% for orthologs, outparalogs, and inparalogs, respectively (P = 0.00003, 0.004, and 0.0003, respectively, n = 5, two-tail t-test), and these annual increases are all significantly different from one another (P<10−11, two-tail Z-test). (PDF) [file pcbi.1002784.s002.pdf]

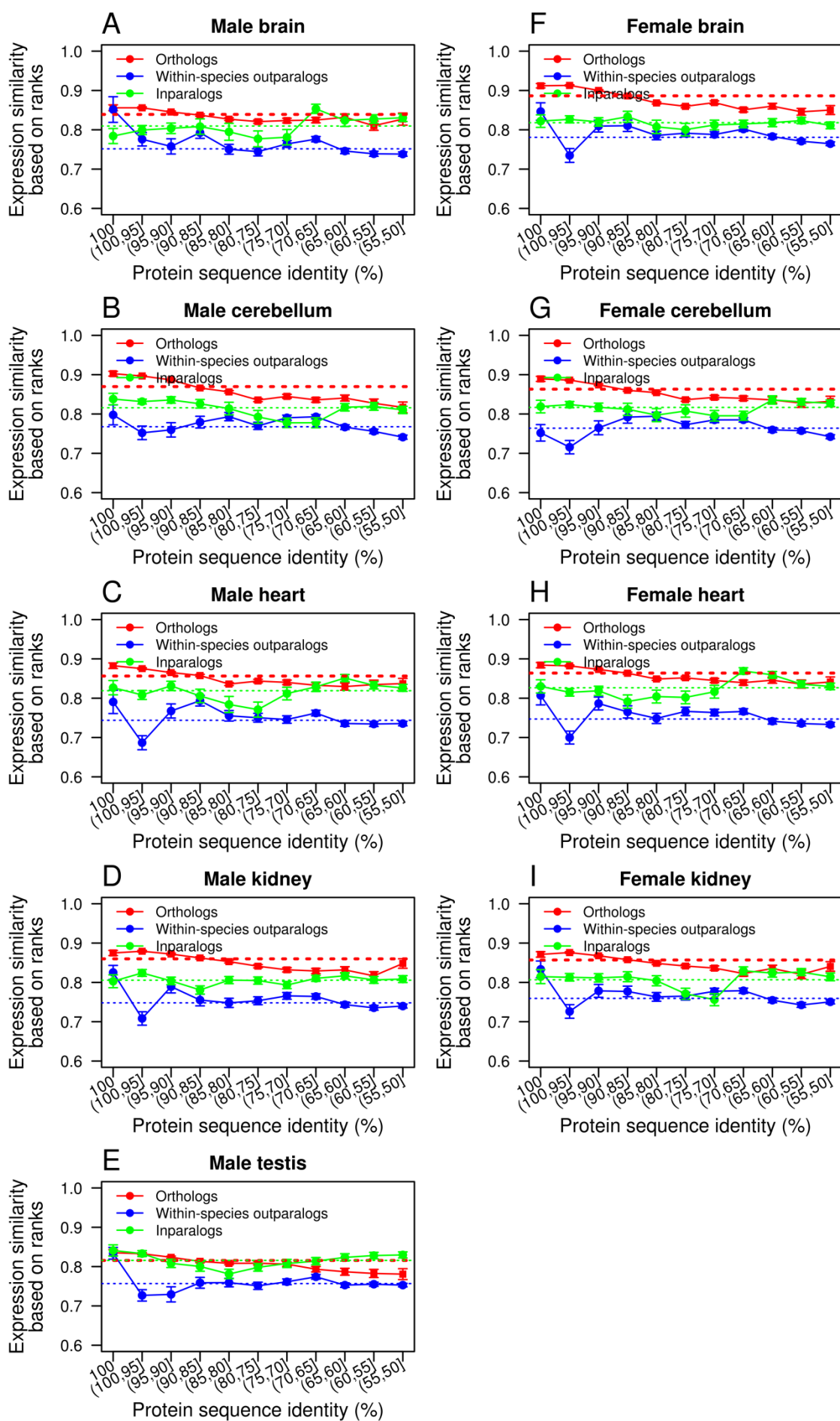

Figure S3

Supplement: Figure S3 — Expression similarities of homologous genes in (A) male brain, (B) male cerebellum, (C) male heart, (D) male kidney, (E) male testis, (F) female brain, (G) female cerebellum, (H) female heart, and (I) female kidney, based on Z-scores. Error bars show one standard error. Each solid line connects the expression similarity values of different bins, whereas the dotted line shows the mean value across all genes in all bins. (PDF) [file pcbi.1002784.s003.pdf]

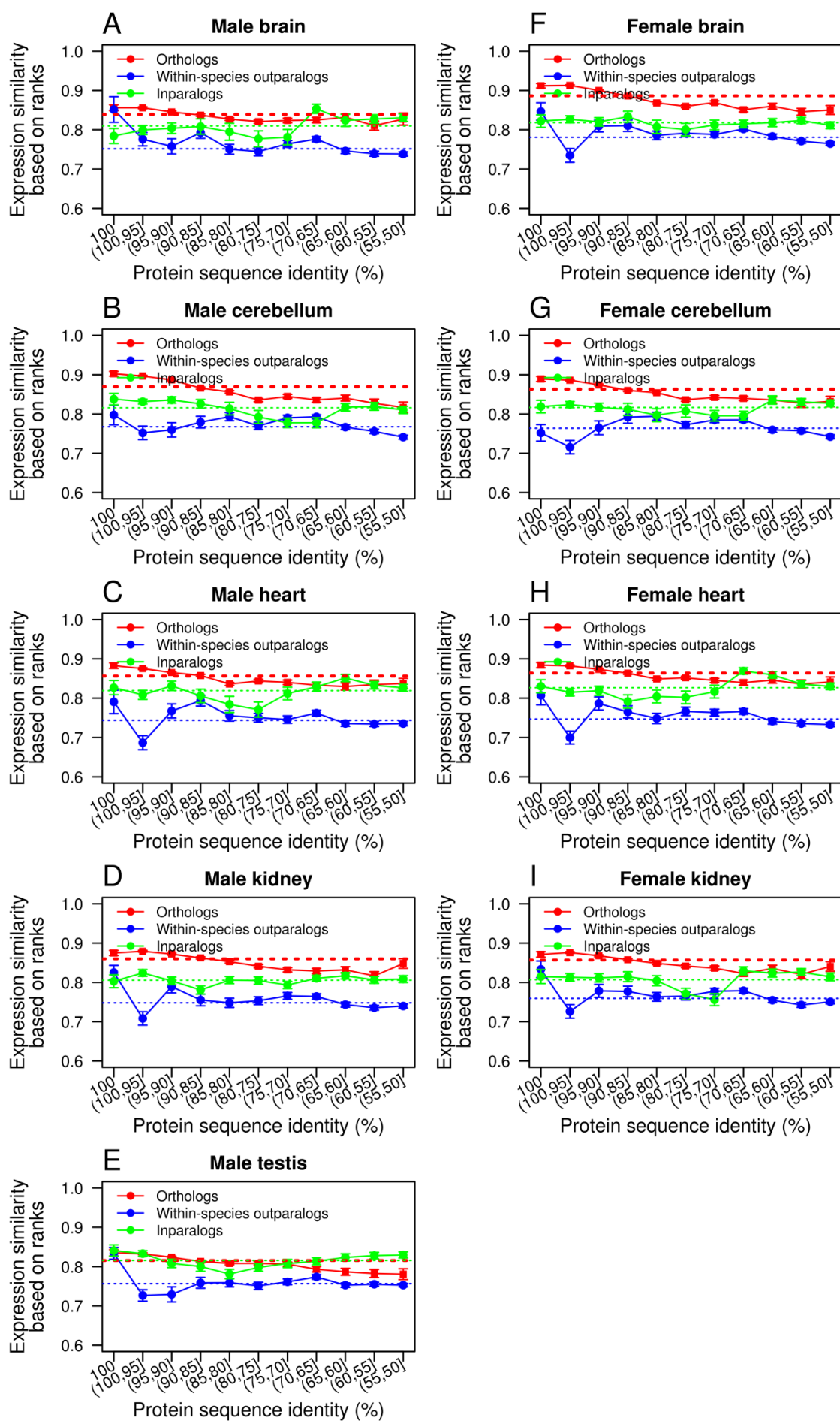

Figure S4

Supplement: Figure S4 — Expression similarities of homologous genes in (A) male brain, (B) male cerebellum, (C) male heart, (D) male kidney, (E) male testis, (F) female brain, (G) female cerebellum, (H) female heart, and (I) female kidney, based on expression ranks. Error bars show one standard error. Each solid line connects the expression similarity values of different bins, whereas the dotted line shows the mean value across all genes in all bins. Note that in male testis the expression similarity between orthologs is almost identical to that between inparalogs (P = 0.49, one-tail Z-test). (PDF) [file pcbi.1002784.s004.pdf]

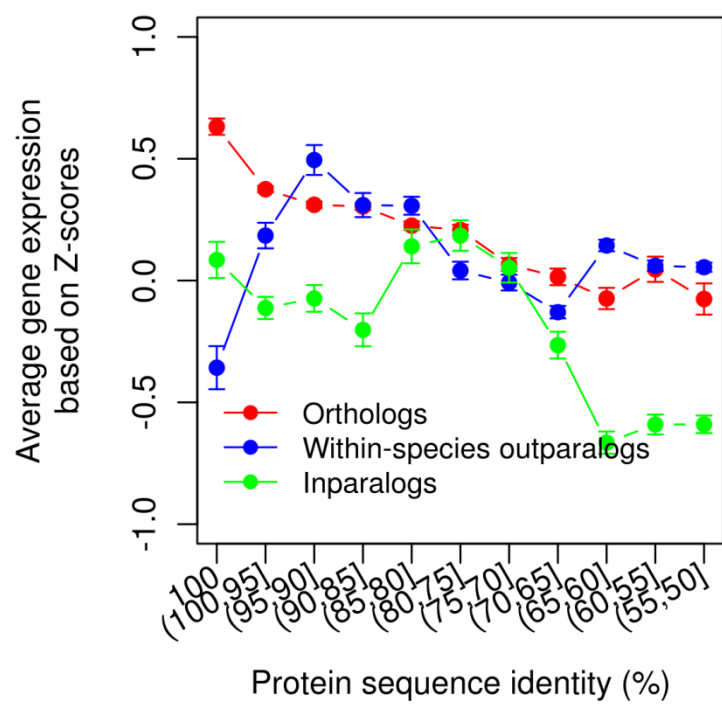

Figure S5

Supplement: Figure S5 — Variations of mean expression levels of orthologs, outparalogs, and inparalogs with different protein sequence identities. (PDF) [file pcbi.1002784.s005.pdf]

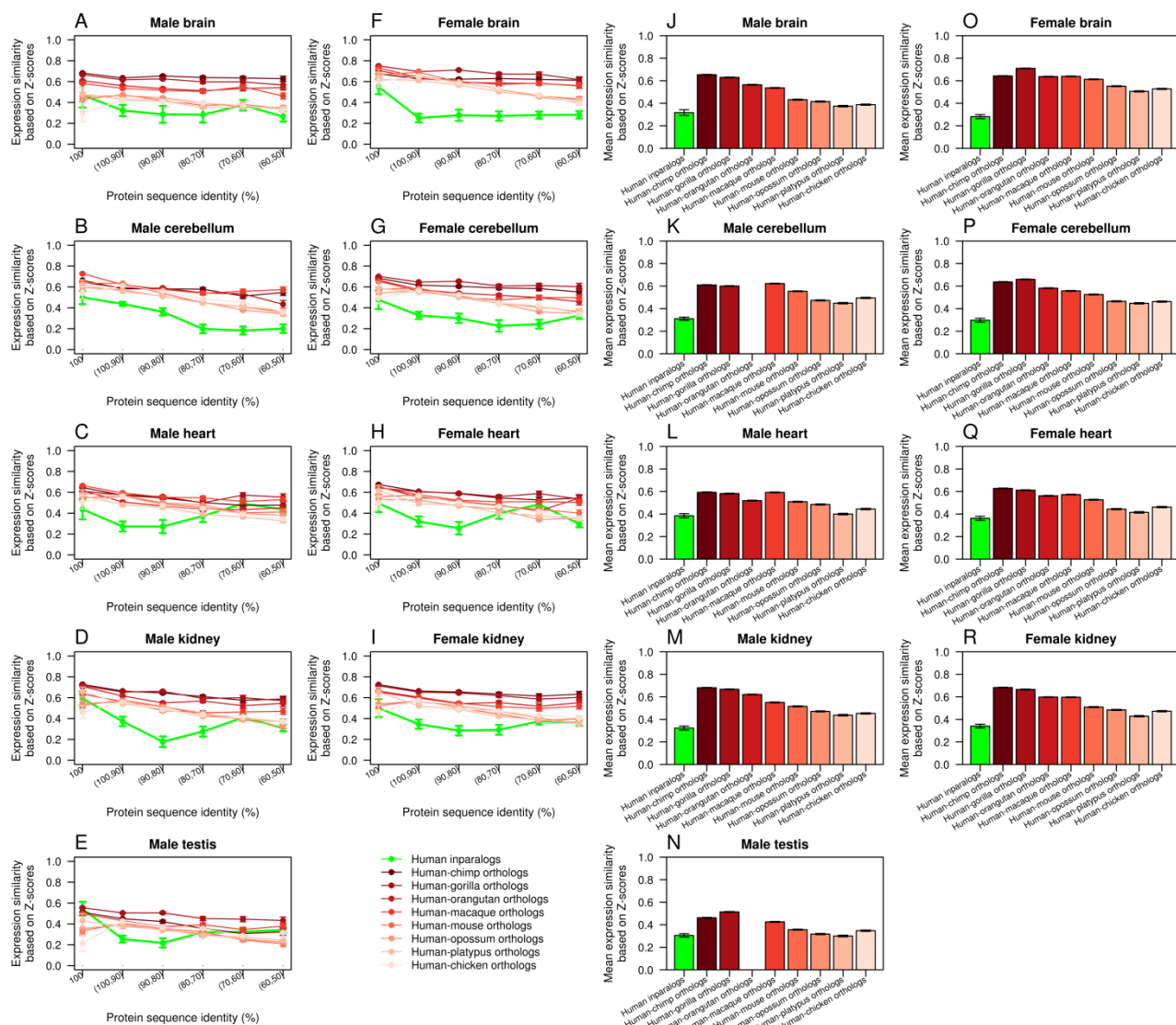

Figure S6

Supplement: Figure S6 — Z-score-based expression similarity of homologous genes from multiple species, examined in nine tissues. Expression similarities of human inparalogs (green dots) are generally lower than those of orthologs between human and multiple species (red dots) for individual protein sequence identity bins in each of the nine tissues: (A) male brain, (B) male cerebellum, (C) male heart, (D) male kidney, (E) male testis, (F) female brain, (G) female cerebellum, (H) female heart, and (I) female kidney. Mean expression similarity of human inparalogs (green bar) and those of orthologs between human and multiple species (red bars) in each of the nine tissues: (J) male brain, (K) male cerebellum, (L) male heart, (M) male kidney, (N) male testis, (O) female brain, (P) female cerebellum, (Q) female heart, and (R) female kidney. For all panels, error bars indicate one standard error. Expression data from certain tissues are not available in some species. (PDF) [file pcbi.1002784.s006.pdf]

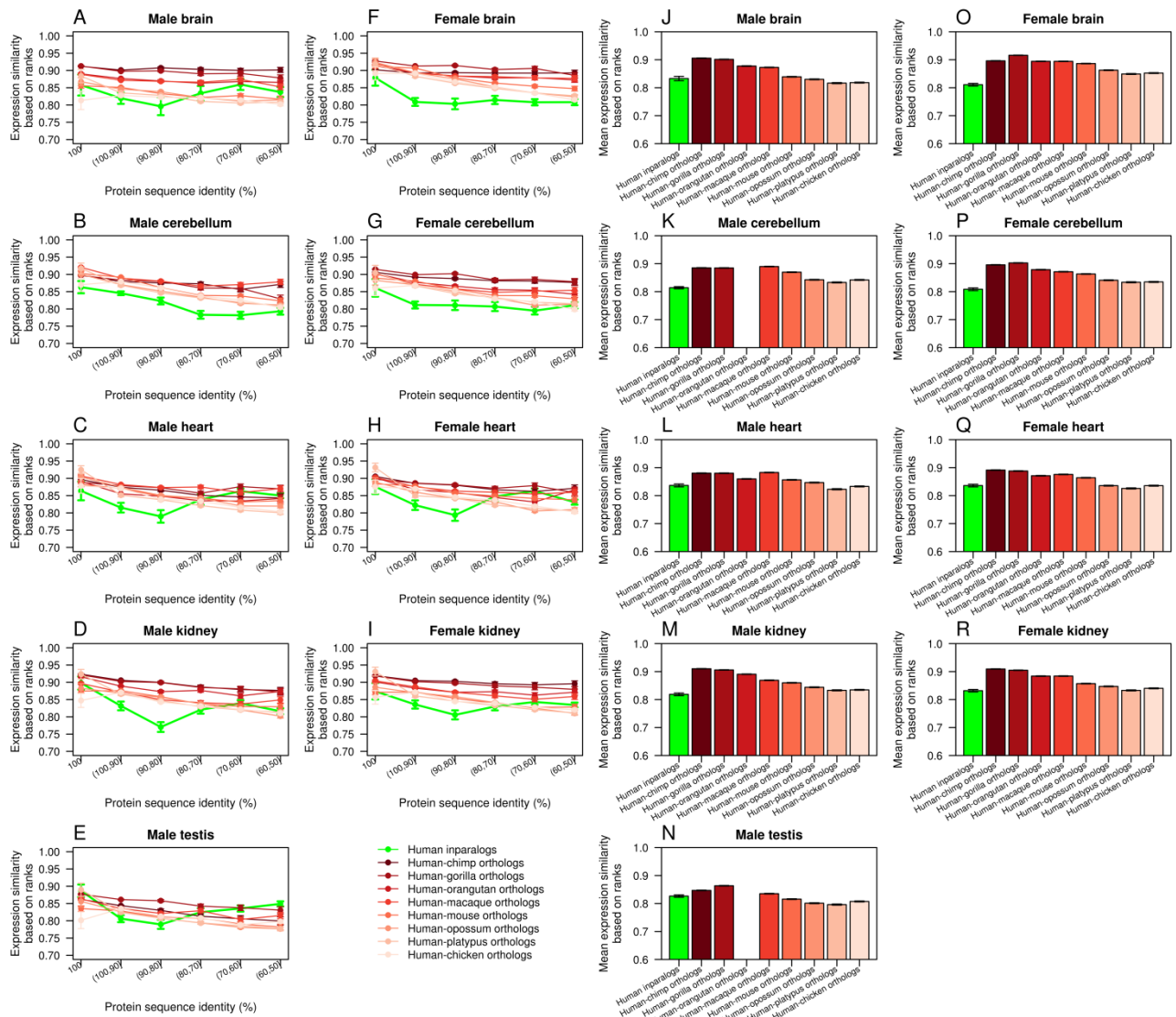

Figure S7

Supplement: Figure S7 — Rank-based expression similarity of homologous genes from multiple species, examined in nine tissues. Expression similarities of human inparalogs (green dots) are generally lower than those of orthologs between human and multiple species (red dots) for individual protein sequence identity bins in each of the nine tissues: (A) male brain, (B) male cerebellum, (C) male heart, (D) male kidney, (E) male testis, (F) female brain, (G) female cerebellum, (H) female heart, and (I) female kidney. Mean expression similarity of human inparalogs (green bar) and those of orthologs between human and multiple species (red bars) in each of the nine tissues: (J) male brain, (K) male cerebellum, (L) male heart, (M) male kidney, (N) male testis, (O) female brain, (P) female cerebellum, (Q) female heart, and (R) female kidney. For all panels, error bars indicate one standard error. Expression data from certain tissues are not available in some species. (PDF) [file pcbi.1002784.s007.pdf]

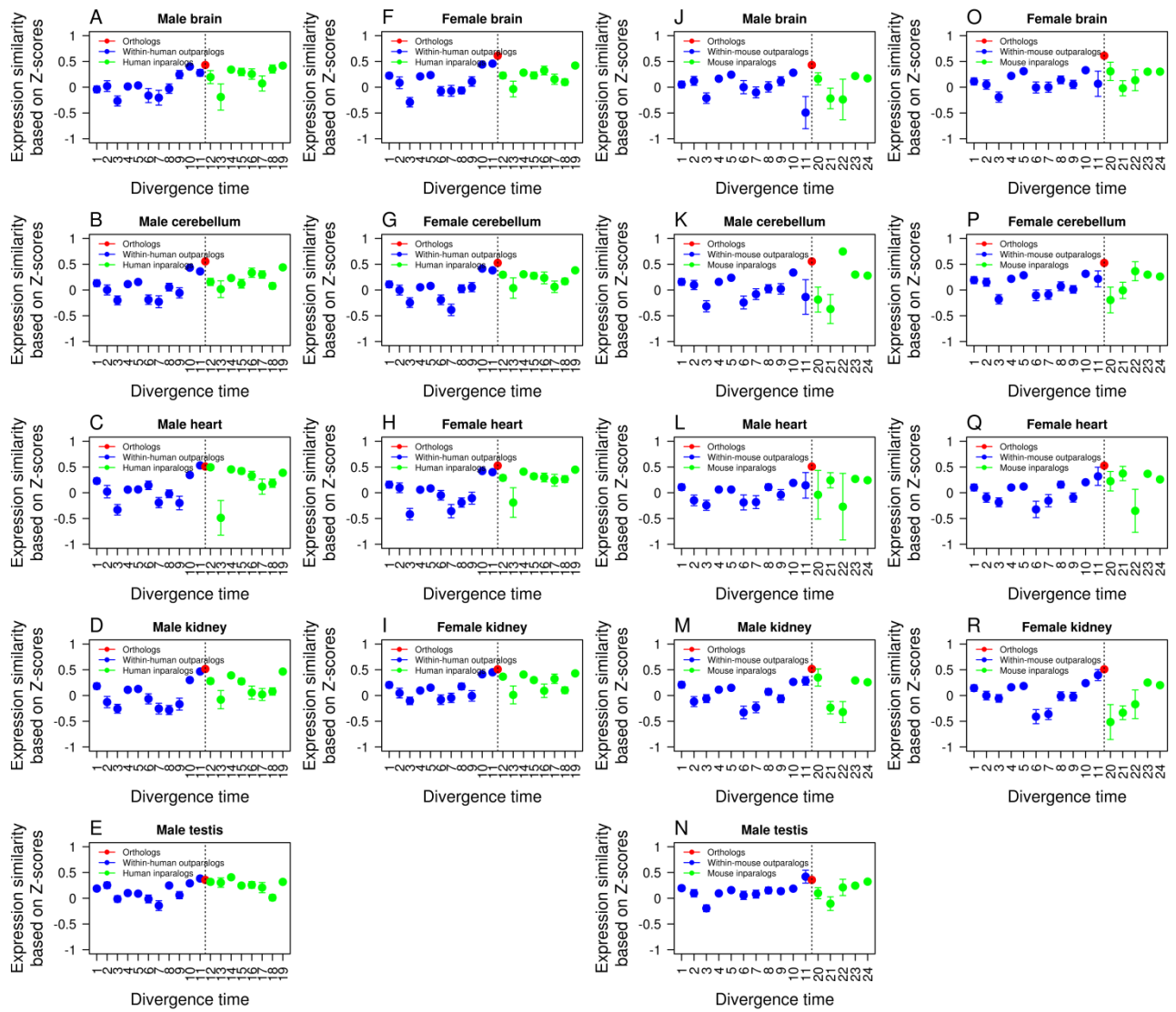

Figure S8

Supplement: Figure S8 — Z-score-based expressions are generally more similar for human-mouse orthologs than (A-I) within-human paralogs and (J-R) within-mouse paralogs across nine tissues. Numbers on the X-axis correspond to the branches in Fig. 5A. Error bars indicate one standard error. The dashed line indicates the human-mouse divergence. (PDF) [file pcbi.1002784.s008.pdf]

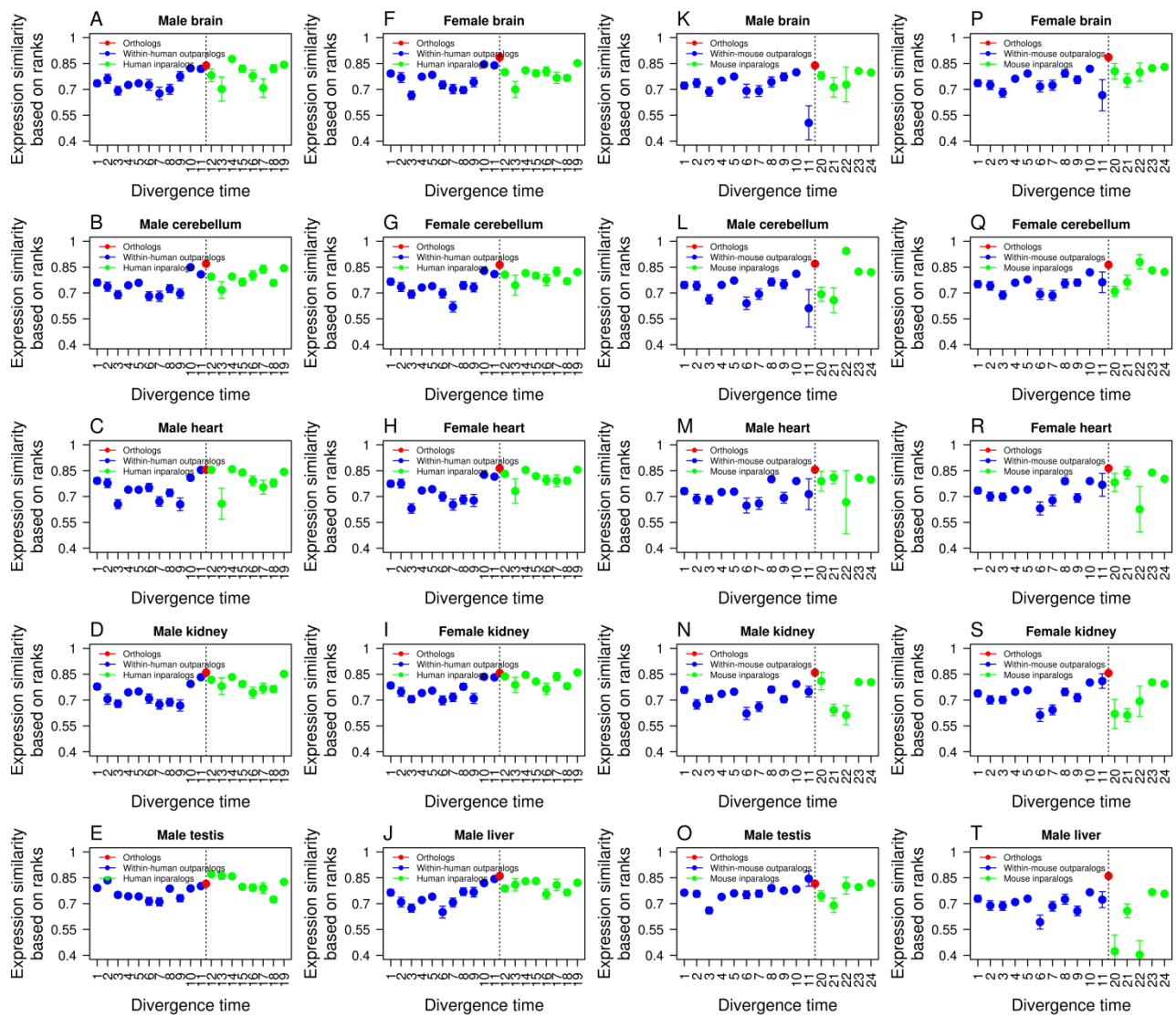

Figure S9

Supplement: Figure S9 — Rank-based expressions are generally more similar for human-mouse orthologs than (A-J) within-human paralogs and (K-T) within-mouse paralogs across 10 tissues. Numbers on the X-axis correspond to the branches in Fig. 5A. Error bars indicate one standard error. The dashed line indicates the human-mouse divergence. (PDF) [file pcbi.1002784.s009.pdf]
