# Supplementary material for: The Ortholog Conjecture Is Untestable by the Current Gene Ontology but Is Supported by RNA Sequencing Data
Source: PLoS Comput Biol. 2012 Nov 29;8(11):e1002784. doi: 10.1371/journal.pcbi.1002784 (PMC3510086; doi:10.1371/journal.pcbi.1002784)
Supplement: Table S1 — Numbers of gene pairs used in the time series analysis (Fig. 1 and Fig. S2). Note that the gene pairs used are the same across all years. (DOC) [file pcbi.1002784.s010.doc]

Table S1. Numbers of gene pairs used in the time series analysis (Fig. 1 and Fig. S2). Note that the gene pairs

used are the same across all years.

| GO aspects | All GO annotations | | |  | Without GO annotations from co-study papers | | |
| --- | --- | --- | --- | --- | --- | --- | --- |
|  | Orthologs | Outparalogs | Inparalogs |  | Orthologs | Outparalogs | Inparalogs |
| Biological process | 287 | 447 | 39 |  | 219 | 204 | 11 |
| Molecular function | 617 | 608 | 55 |  | 482 | 251 | 14 |
| Cellular component | 331 | 401 | 35 |  | 252 | 143 | 6 |
