# Supplementary material for: The Ortholog Conjecture Is Untestable by the Current Gene Ontology but Is Supported by RNA Sequencing Data
Source: PLoS Comput Biol. 2012 Nov 29;8(11):e1002784. doi: 10.1371/journal.pcbi.1002784 (PMC3510086; doi:10.1371/journal.pcbi.1002784)
Supplement: Table S2 — Human and mouse homologous genes with identical protein sequences in co-study papers. (DOC) [file pcbi.1002784.s011.doc]

Table S2. Human and mouse homologous genes with identical protein sequences in co-study papers

| **PubMed ID** | **Ensembl gene ID** | **GO term accession** | **GO category** | **GO term description** |  |
| --- | --- | --- | --- | --- | --- |
| PMID:11113207 | ENSG00000168772 | GO:0031410 | cellular_component | cytoplasmic vesicle |  |
|  |  | GO:0030178 | biological_process | negative regulation of Wnt receptor signaling pathway |  |
|  |  | GO:0030165 | molecular_function | PDZ domain binding |  |
|  |  | GO:0005737 | cellular_component | cytoplasm |  |
|  | ENSMUSG00000044365 | GO:0016055 | biological_process | Wnt receptor signaling pathway |  |
|  |  | GO:0016023 | cellular_component | cytoplasmic membrane-bounded vesicle |  |
|  |  | GO:0005737 | cellular_component | cytoplasm |  |
| PMID:7890768 | ENSG00000121989 | GO:0048186 | molecular_function | inhibin beta-A binding |  |
|  | ENSMUSG00000052155 | GO:0048186 | molecular_function | inhibin beta-A binding |  |
|  |  | GO:0005515 | molecular_function | protein binding |  |
| PMID:11870224 | ENSG00000167193 | GO:0046875 | molecular_function | ephrin receptor binding |  |
|  |  | GO:0032319 | biological_process | regulation of Rho GTPase activity |  |
|  |  | GO:0048013 | biological_process | ephrin receptor signaling pathway |  |
|  |  | GO:0032956 | biological_process | regulation of actin cytoskeleton organization |  |
|  | ENSMUSG00000017776 | GO:0046875 | molecular_function | ephrin receptor binding |  |
| PMID:17581632 | ENSG00000104408 | GO:0006413 | biological_process | translational initiation |  |
|  |  | GO:0003743 | molecular_function | translation initiation factor activity |  |
|  |  | GO:0005852 | cellular_component | eukaryotic translation initiation factor 3 complex |  |
|  | ENSMUSG00000022336 | GO:0006413 | biological_process | translational initiation |  |
|  |  | GO:0003743 | molecular_function | translation initiation factor activity |  |
|  |  | GO:0005852 | cellular_component | eukaryotic translation initiation factor 3 complex |  |
| PMID:17110330 | ENSG00000172977 | GO:0005515 | molecular_function | protein binding |  |
|  | ENSMUSG00000024926 | GO:0005515 | molecular_function | protein binding |  |
| PMID:9173976 | ENSG00000087191 | GO:0005515 | molecular_function | protein binding |  |
|  |  | GO:0008134 | molecular_function | transcription factor binding |  |
|  | ENSMUSG00000020708 | GO:0045892 | biological_process | negative regulation of transcription, DNA-dependent |  |
|  |  | GO:0005515 | molecular_function | protein binding |  |
| PMID:10980193 | ENSG00000122644 | GO:0005634 | cellular_component | nucleus |  |
|  |  | GO:0005730 | cellular_component | nucleolus |  |
|  |  | GO:0005515 | molecular_function | protein binding |  |
|  |  | GO:0005525 | molecular_function | GTP binding |  |
|  | ENSMUSG00000047446 | GO:0005634 | cellular_component | nucleus |  |
| PMID:10913114 | ENSG00000127337 | GO:0005654 | cellular_component | nucleoplasm |  |
|  |  | GO:0008022 | molecular_function | protein C-terminus binding |  |
|  | ENSMUSG00000020171 | GO:0005654 | cellular_component | nucleoplasm |  |
|  |  | GO:0005515 | molecular_function | protein binding |  |
| PMID:14722116 | ENSG00000177885 | GO:0005515 | molecular_function | protein binding |  |
|  | ENSMUSG00000059923 | GO:0005515 | molecular_function | protein binding |  |
| PMID:18234692 | ENSG00000168610 | GO:0005634 | cellular_component | nucleus |  |
|  |  | GO:0005515 | molecular_function | protein binding |  |
|  |  | GO:0006606 | biological_process | protein import into nucleus |  |
|  |  | GO:0005737 | cellular_component | cytoplasm |  |
|  | ENSMUSG00000004040 | GO:0005515 | molecular_function | protein binding |  |
| PMID:17178831 | ENSG00000134138 | GO:0043565 | molecular_function | sequence-specific DNA binding |  |
|  | ENSMUSG00000027210 | GO:0005634 | cellular_component | nucleus |  |
|  |  | GO:0045944 | biological_process | positive regulation of transcription from RNA polymerase II promoter |  |
|  |  | GO:0006355 | biological_process | regulation of transcription, DNA-dependent |  |
|  |  | GO:0043565 | molecular_function | sequence-specific DNA binding |  |
|  |  | GO:0003700 | molecular_function | sequence-specific DNA binding transcription factor activity |  |
| PMID:15004007 | ENSG00000087095 | GO:0007179 | biological_process | transforming growth factor beta receptor signaling pathway |  |
|  | ENSMUSG00000017376 | GO:0033136 | biological_process | serine phosphorylation of STAT3 protein |  |
|  |  | GO:0004674 | molecular_function | protein serine/threonine kinase activity |  |
|  |  | GO:0042169 | molecular_function | SH2 domain binding |  |
| PMID:7555706 | ENSG00000008196 | GO:0000981 | molecular_function | sequence-specific DNA binding RNA polymerase II transcription factor activity |  |
|  |  | GO:0005515 | molecular_function | protein binding |  |
|  |  | GO:0003713 | molecular_function | transcription coactivator activity |  |
|  |  | GO:0000979 | molecular_function | RNA polymerase II core promoter sequence-specific DNA binding |  |
|  |  | GO:0045944 | biological_process | positive regulation of transcription from RNA polymerase II promoter |  |
|  |  | GO:0006366 | biological_process | transcription from RNA polymerase II promoter |  |
|  | ENSMUSG00000025927 | GO:0003713 | molecular_function | transcription coactivator activity |  |
|  |  | GO:0045944 | biological_process | positive regulation of transcription from RNA polymerase II promoter |  |
|  |  | GO:0001077 | molecular_function | RNA polymerase II core promoter proximal region sequence-specific DNA binding transcription factor activity involved in positive regulation of transcription |  |
| PMID:11146101 | ENSG00000170296 | GO:0005515 | molecular_function | protein binding |  |
|  | ENSMUSG00000018567 | GO:0005790 | cellular_component | smooth endoplasmic reticulum |  |
|  |  | GO:0005794 | cellular_component | Golgi apparatus |  |
|  |  | GO:0005764 | cellular_component | lysosome |  |
| PMID:14726512 | ENSG00000134809 | GO:0005515 | molecular_function | protein binding |  |
|  |  | GO:0005743 | cellular_component | mitochondrial inner membrane |  |
|  |  | GO:0042719 | cellular_component | mitochondrial intermembrane space protein transporter complex |  |
|  | ENSMUSG00000027076 | GO:0042719 | cellular_component | mitochondrial intermembrane space protein transporter complex |  |
|  |  | GO:0045039 | biological_process | protein import into mitochondrial inner membrane |  |
| PMID:10369680 | ENSG00000121481 | GO:0005515 | molecular_function | protein binding |  |
|  | ENSMUSG00000026484 | GO:0005515 | molecular_function | protein binding |  |
| PMID:16525025 | ENSG00000169750 | GO:0030426 | cellular_component | growth cone |  |
|  |  | GO:0043025 | cellular_component | neuronal cell body |  |
|  |  | GO:0043005 | cellular_component | neuron projection |  |
|  |  | GO:0031941 | cellular_component | filamentous actin |  |
|  | ENSMUSG00000018012 | GO:0031175 | biological_process | neuron projection development |  |
| PMID:11438699 | ENSG00000055163 | GO:0019717 | cellular_component | synaptosome |  |
|  |  | GO:0005515 | molecular_function | protein binding |  |
|  |  | GO:0005737 | cellular_component | cytoplasm |  |
|  | ENSMUSG00000020340 | GO:0005515 | molecular_function | protein binding |  |
| PMID:20592023 | ENSG00000007372 | GO:0000981 | molecular_function | sequence-specific DNA binding RNA polymerase II transcription factor activity |  |
|  |  | GO:0010628 | biological_process | positive regulation of gene expression |  |
|  |  | GO:0000790 | cellular_component | nuclear chromatin |  |
|  |  | GO:0045893 | biological_process | positive regulation of transcription, DNA-dependent |  |
|  |  | GO:0006366 | biological_process | transcription from RNA polymerase II promoter |  |
|  |  | GO:0006357 | biological_process | regulation of transcription from RNA polymerase II promoter |  |
|  |  | GO:0000979 | molecular_function | RNA polymerase II core promoter sequence-specific DNA binding |  |
|  |  | GO:0003322 | biological_process | pancreatic A cell development |  |
|  | ENSMUSG00000027168 | GO:0000981 | molecular_function | sequence-specific DNA binding RNA polymerase II transcription factor activity |  |
|  |  | GO:0003322 | biological_process | pancreatic A cell development |  |
|  |  | GO:0000979 | molecular_function | RNA polymerase II core promoter sequence-specific DNA binding |  |
|  |  | GO:0010628 | biological_process | positive regulation of gene expression |  |
|  |  | GO:0000790 | cellular_component | nuclear chromatin |  |
|  |  | GO:0045893 | biological_process | positive regulation of transcription, DNA-dependent |  |
|  |  | GO:0006366 | biological_process | transcription from RNA polymerase II promoter |  |
| PMID:16525503 | ENSG00000165280 | GO:0005515 | molecular_function | protein binding |  |
|  | ENSMUSG00000028452 | GO:0005515 | molecular_function | protein binding |  |
| PMID:19154719 | ENSG00000198435 | GO:0045746 | biological_process | negative regulation of Notch signaling pathway |  |
|  |  | GO:0090263 | biological_process | positive regulation of canonical Wnt receptor signaling pathway |  |
|  | ENSMUSG00000078202 | GO:0002043 | biological_process | blood vessel endothelial cell proliferation involved in sprouting angiogenesis |  |
|  |  | GO:0001569 | biological_process | patterning of blood vessels |  |
|  |  | GO:0001938 | biological_process | positive regulation of endothelial cell proliferation |  |
|  |  | GO:0022407 | biological_process | regulation of cell-cell adhesion |  |
|  |  | GO:0002040 | biological_process | sprouting angiogenesis |  |
| PMID:11804788 | ENSG00000143621 | GO:0003677 | molecular_function | DNA binding |  |
|  | ENSMUSG00000001016 | GO:0005634 | cellular_component | nucleus |  |
|  |  | GO:0005730 | cellular_component | nucleolus |  |
| PMID:15601820 | ENSG00000154582 | GO:0005515 | molecular_function | protein binding |  |
|  | ENSMUSG00000079658 | GO:0005515 | molecular_function | protein binding |  |
| PMID:11302691 | ENSG00000215021 | GO:0005515 | molecular_function | protein binding |  |
|  | ENSMUSG00000004264 | GO:0005743 | cellular_component | mitochondrial inner membrane |  |
| PMID:20005108 | ENSG00000006451 | GO:0051665 | biological_process | membrane raft localization |  |
|  |  | GO:0017157 | biological_process | regulation of exocytosis |  |
|  | ENSMUSG00000008859 | GO:0051665 | biological_process | membrane raft localization |  |
|  |  | GO:0017157 | biological_process | regulation of exocytosis |  |
| PMID:18816825 | ENSG00000082014 | GO:0071564 | cellular_component | npBAF complex |  |
|  |  | GO:0005634 | cellular_component | nucleus |  |
|  |  | GO:0003407 | biological_process | neural retina development |  |
|  |  | GO:0002052 | biological_process | positive regulation of neuroblast proliferation |  |
|  | ENSMUSG00000028949 | GO:0005634 | cellular_component | nucleus |  |
| PMID:20159109 | ENSG00000155961 | GO:0005794 | cellular_component | Golgi apparatus |  |
|  | ENSMUSG00000031202 | GO:0005794 | cellular_component | Golgi apparatus |  |
|  |  | GO:0050808 | biological_process | synapse organization |  |
|  |  | GO:0016192 | biological_process | vesicle-mediated transport |  |
| PMID:15722337 | ENSG00000108953 | GO:0005515 | molecular_function | protein binding |  |
|  | ENSMUSG00000020849 | GO:0005515 | molecular_function | protein binding |  |
| PMID:11595183 | ENSG00000155849 | GO:0016601 | biological_process | Rac protein signal transduction |  |
|  |  | GO:0006928 | biological_process | cellular component movement |  |
|  |  | GO:0006911 | biological_process | phagocytosis, engulfment |  |
|  |  | GO:0030036 | biological_process | actin cytoskeleton organization |  |
|  |  | GO:0005886 | cellular_component | plasma membrane |  |
|  |  | GO:0005737 | cellular_component | cytoplasm |  |
|  | ENSMUSG00000041112 | GO:0030029 | biological_process | actin filament-based process |  |
|  |  | GO:0006909 | biological_process | phagocytosis |  |
| PMID:18339854 | ENSG00000119048 | GO:0005634 | cellular_component | nucleus |  |
|  |  | GO:0006513 | biological_process | protein monoubiquitination |  |
|  |  | GO:0033522 | biological_process | histone H2A ubiquitination |  |
|  |  | GO:0050821 | biological_process | protein stabilization |  |
|  |  | GO:0005737 | cellular_component | cytoplasm |  |
|  |  | GO:0004842 | molecular_function | ubiquitin-protein ligase activity |  |
|  |  | GO:0000209 | biological_process | protein polyubiquitination |  |
|  | ENSMUSG00000020390 | GO:0060070 | biological_process | canonical Wnt receptor signaling pathway |  |
| PMID:12949260 | ENSG00000168036 | GO:0003713 | molecular_function | transcription coactivator activity |  |
|  |  | GO:0045893 | biological_process | positive regulation of transcription, DNA-dependent |  |
|  | ENSMUSG00000006932 | GO:0003713 | molecular_function | transcription coactivator activity |  |
|  |  | GO:0045893 | biological_process | positive regulation of transcription, DNA-dependent |  |
| PMID:12689335* | ENSMUSG00000073293 | GO:0008152 | biological_process | metabolic process |  |
|  |  | GO:0008486 | molecular_function | diphosphoinositol-polyphosphate diphosphatase activity |  |
|  | ENSMUSG00000073295 | GO:0008152 | biological_process | metabolic process |  |
|  |  | GO:0008486 | molecular_function | diphosphoinositol-polyphosphate diphosphatase activity |  |
| PMID:8524813* | ENSMUSG00000052305 | GO:0048821 | biological_process | erythrocyte development |  |
|  |  | GO:0005833 | cellular_component | hemoglobin complex |  |
|  | ENSMUSG00000073940 | GO:0048821 | biological_process | erythrocyte development |  |
|  |  | GO:0005833 | cellular_component | hemoglobin complex |  |
| PMID:7550311* | ENSMUSG00000069917 | GO:0048821 | biological_process | erythrocyte development |  |
|  |  | GO:0001701 | biological_process | in utero embryonic development |  |
|  | ENSMUSG00000069919 | GO:0048821 | biological_process | erythrocyte development |  |
|  |  | GO:0001701 | biological_process | in utero embryonic development |  |
| PMID:14701760* | ENSMUSG00000090877 | GO:0006281 | biological_process | DNA repair |  |
|  |  | GO:0009408 | biological_process | response to heat |  |
|  |  | GO:0000723 | biological_process | telomere maintenance |  |
|  | ENSMUSG00000091971 | GO:0006281 | biological_process | DNA repair |  |
|  |  | GO:0009408 | biological_process | response to heat |  |
|  |  | GO:0000723 | biological_process | telomere maintenance |  |
| PMID:18614015* | ENSMUSG00000076436 | GO:0005739 | cellular_component | mitochondrion |  |
|  | ENSMUSG00000076438 | GO:0005739 | cellular_component | mitochondrion |  |
| PMID:2469574** | ENSMUSG00000001175 | GO:0007049 | biological_process | cell cycle |  |
|  | ENSMUSG00000036438 | GO:0007049 | biological_process | cell cycle |  |
| PMID:2469574** | ENSMUSG00000001175 | GO:0007049 | biological_process | cell cycle |  |
|  | ENSMUSG00000019370 | GO:0007049 | biological_process | cell cycle |  |
| PMID:2469574** | ENSMUSG00000019370 | GO:0007049 | biological_process | cell cycle |  |
|  | ENSMUSG00000036438 | GO:0007049 | biological_process | cell cycle |  |
| PMID:19103752** | ENSMUSG00000023004 | GO:0005881 | cellular_component | cytoplasmic microtubule |  |
|  | ENSMUSG00000072235 | GO:0005881 | cellular_component | cytoplasmic microtubule |  |

*, Co-study papers of inparalogs; **, co-study papers of within-species outparalogs; the rest are orthologs.
