# Supplementary material for: The Ortholog Conjecture Is Untestable by the Current Gene Ontology but Is Supported by RNA Sequencing Data
Source: PLoS Comput Biol. 2012 Nov 29;8(11):e1002784. doi: 10.1371/journal.pcbi.1002784 (PMC3510086; doi:10.1371/journal.pcbi.1002784)
Supplement: Table S3 — Numbers of gene pairs used at each level (bin) of sequence identity in human and mouse RNA-Seq analysis (Fig. 3). (DOC) [file pcbi.1002784.s012.doc]

Table S3. Numbers of gene pairs used at each level (bin) of sequence identity in human and

mouse RNA-Seq analysis (Fig. 3).

| Identity |  | Liver | | |  | All 10 tissues | | |
| --- | --- | --- | --- | --- | --- | --- | --- | --- |
|  |  | Orthologs | Outparalogs | Inparalogs |  | Orthologs | Outparalogs | Inparalogs |
| 100 |  | 326 | 65 | 93 |  | 305 | 18 | 58 |
| (100,95] |  | 2533 | 195 | 313 |  | 2383 | 98 | 103 |
| (95,90] |  | 2339 | 144 | 261 |  | 2169 | 90 | 66 |
| (90,85] |  | 2015 | 203 | 159 |  | 1806 | 130 | 42 |
| (85,80] |  | 1550 | 319 | 173 |  | 1303 | 236 | 47 |
| (80,75] |  | 1080 | 398 | 218 |  | 880 | 284 | 63 |
| (75,70] |  | 705 | 571 | 235 |  | 529 | 375 | 53 |
| (70,65] |  | 495 | 939 | 266 |  | 353 | 548 | 85 |
| (65,60] |  | 309 | 1018 | 223 |  | 205 | 720 | 80 |
| (60,55] |  | 214 | 1194 | 242 |  | 146 | 845 | 113 |
| (55,50] |  | 130 | 1493 | 231 |  | 87 | 1055 | 132 |
