# Supplementary material for: The Ortholog Conjecture Is Untestable by the Current Gene Ontology but Is Supported by RNA Sequencing Data
Source: PLoS Comput Biol. 2012 Nov 29;8(11):e1002784. doi: 10.1371/journal.pcbi.1002784 (PMC3510086; doi:10.1371/journal.pcbi.1002784)
Supplement: Table S4 — Numbers of gene pairs used at each level (bin) of sequence identity in the multiple-species RNA-Seq analysis (Fig. 4). (DOC) [file pcbi.1002784.s013.doc]

Table S4. Numbers of gene pairs used at each level (bin) of sequence identity in the multiple-species

RNA-Seq analysis (Fig. 4).

| Identity | Inparalogs |  | Orthologs | | | | | | | |
| --- | --- | --- | --- | --- | --- | --- | --- | --- | --- | --- |
|  | Human |  | Chimp | Gorilla | Orangutan | Macaque | Mouse | Opossum | Platypus | Chicken |
| 100 | 28 |  | 4923 | 3364 | 2247 | 1423 | 326 | 110 | 43 | 53 |
| (100,95] | 189 |  | 6687 | 6898 | 8389 | 8093 | 4872 | 2020 | 759 | 1145 |
| (90,80] | 152 |  | 885 | 1106 | 1148 | 1477 | 3565 | 2569 | 1239 | 1764 |
| (80,70] | 156 |  | 419 | 451 | 516 | 622 | 1785 | 2328 | 1422 | 1852 |
| (70,60] | 239 |  | 238 | 264 | 300 | 373 | 804 | 1510 | 1359 | 1639 |
| (60,50] | 224 |  | 141 | 147 | 208 | 243 | 344 | 850 | 1078 | 1131 |
