# Supplementary figures and images for: Correction: The Ortholog Conjecture Is Untestable by the Current Gene Ontology but Is Supported by RNA Sequencing Data
Source: PLoS Comput Biol. 2013 Jan 31;9(1):10.1371/annotation/6b5adbad-8944-4ab4-acd9-ac6f0d3e624e. doi: 10.1371/annotation/6b5adbad-8944-4ab4-acd9-ac6f0d3e624e (PMC4019461; doi:10.1371/annotation/6b5adbad-8944-4ab4-acd9-ac6f0d3e624e)

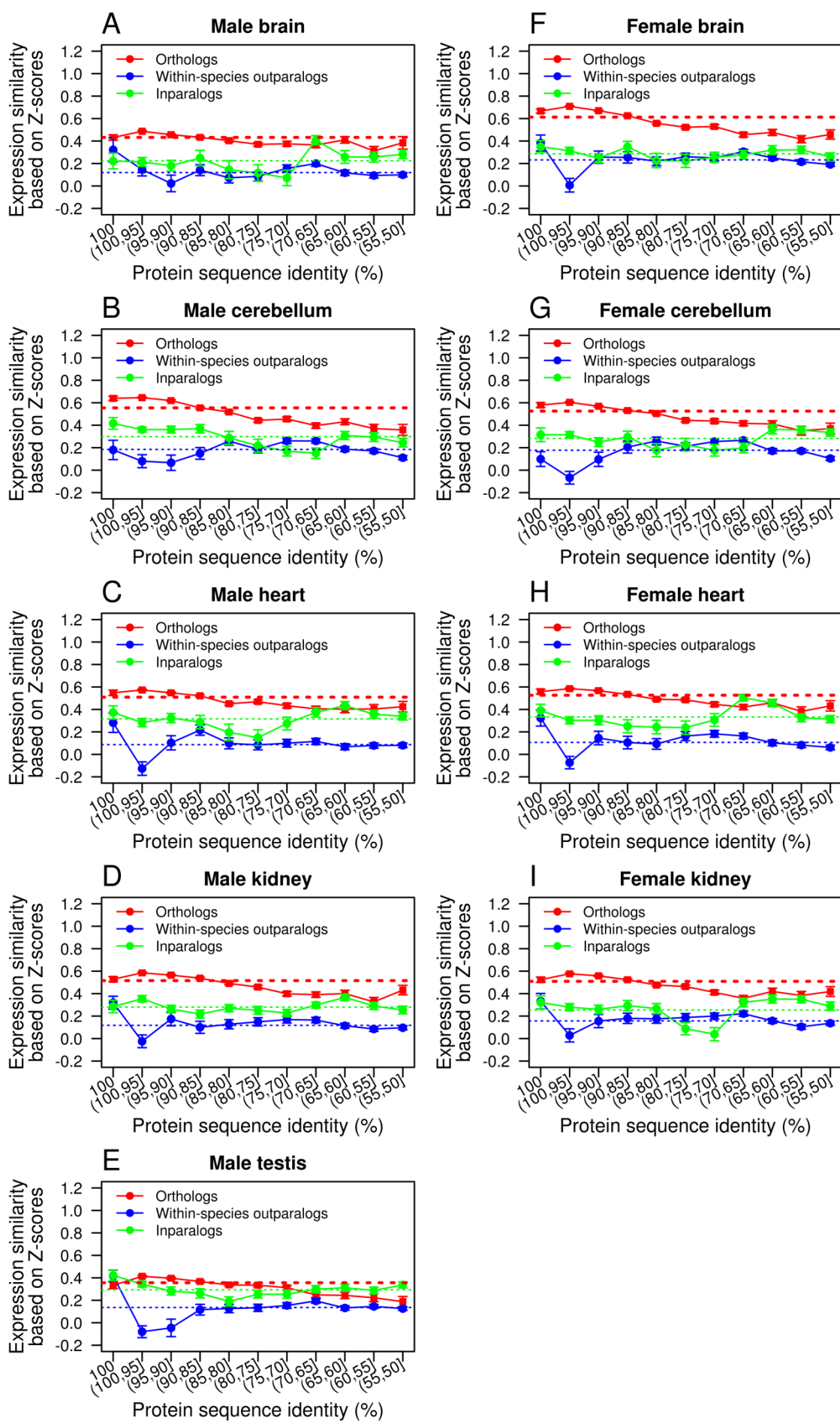

Figure S3

Supplement: Supplementary file 1 [file pcbi.6b5adbad-8944-4ab4-acd9-ac6f0d3e624e.s001.pdf]
